# Whole-Genome Sequencing and Target Validation Analysis of Müllerian Adenosarcoma: A Tumor With Complex but Specific Genetic Alterations

**DOI:** 10.3389/fonc.2020.00538

**Published:** 2020-04-15

**Authors:** Yanli Ban, Jean V. Fischer, Kruti P. Maniar, Haiyang Guo, Chang Zeng, Yinuo Li, Qing Zhang, Xinkun Wang, Wei Zhang, Serdar E. Bulun, Jian-Jun Wei

**Affiliations:** ^1^Department of Pathology, Northwestern University Feinberg School of Medicine, Chicago, IL, United States; ^2^Department of Obstetrics and Gynecology, Qilu Hospital, Shandong University, Jinan, China; ^3^Department of Biology, Princess Margaret Cancer Centre, Ontario Cancer Institute, Toronto, ON, Canada; ^4^Department of Preventive Medicine, Northwestern University Feinberg School of Medicine, Chicago, IL, United States; ^5^Department of Obstetrics and Gynecology, Northwestern University Feinberg School of Medicine, Chicago, IL, United States

**Keywords:** Müllerian adenosarcoma, whole-genome sequencing, copy number variation, pathway analysis, target gene mutations

## Abstract

Mullerian adenosarcoma (MAS) is a biphasic tumor with malignant stroma. It is most commonly of endometrial origin but occasionally originates in the cervix, ovary, or other pelvic/peritoneal sites. The typical MAS is low grade with an indolent clinical course; however, tumors with sarcomatous overgrowth (SO) or a high-grade sarcoma tend to be aggressive. Tumor etiology is largely unknown. To better understand the global genome alterations and gene mutations in MAS, whole-genome sequencing (WGS) and target validation analysis were performed. MAS showed remarkable chromosome (chr) copy number variation (CNV), specifically, gains in chr 1q, 5p, 12p, 12q, and 17q and losses in chr 3p, 3q, 9p, and 11q. Gain of chr 12q13-15 was present in 50% of cases. The selected gene products in gain regions were upregulated as measured by immunohistochemistry. HMGA2 overexpression was significantly correlated with SO. While the structural variation (SV) rate was relatively low overall, a disproportionally high rate of break-ends at chr 7 was noted involving 6 in-frame rearrangement fusion genes. Among 40 frequently mutated genes detected by WGS and validated in 29 MAS by next generation sequencing (NGS), *KMT2C*, and *BCOR* were frequently seen in MAS both with and without SO, while *MAGEC1* and *KDM6B* were strongly associated with SO. Overall, a higher rate of frequently mutated genes was found in MAS with SO (33%) than MAS without (11%). This study uncovers the complex and specific genetic alterations in this malignancy. The findings provide a tool for future investigation of these molecular changes in tumorigenesis and target therapies.

## Introduction

Müllerian adenosarcoma (MAS) is a biphasic tumor accounting for 5–7% of all uterine sarcomas (1–3). It consists of a malignant stromal component admixed with a benign glandular component. MAS is most commonly of endometrial origin, but infrequently arises from cervix, ovary, or other pelvic/peritoneal sites (4–6). Typical MAS is low grade; however, two histologic features with prognostic significance have been described: MAS with stromal overgrowth [SO: pure sarcoma component comprising ≥25% of the tumor (7)] and high-grade sarcomatous transformation (8). SO is present in 33–53% of MAS, and is associated with older age, higher rates of recurrence and death, higher stage at presentation, and lower overall survival (6, 7, 9–13). Treatment of MAS typically consists of hysterectomy; occasionally, more conservative resection is performed (4, 10).

The pathogenesis of MAS is not well-understood. The genomic landscape and molecular pathogenesis of MAS and its variants remain unclear. Prior studies have employed a variety of methods, including targeted sequencing using cancer-related gene panels, whole-exome sequencing, fluorescence *in situ* hybridization, conventional cytogenetics, and immunohistochemistry (IHC), to evaluate the molecular changes in varied sample size of MAS cases (8, 9, 14–17). Some commonalities have been identified in terms of copy number variations (CNVs) and mutations, but considerable heterogeneity is present, and no dominant or driver mutation has been identified. Studies to date suggest that MAS and its variants are genetically heterogeneous and CNVs are more frequent with SO (9, 15). Unlike other uterine sarcomas, MAS does not demonstrate specific non-random chromosomal translocations or fusion gene abnormalities.

To better explore the global genome alterations in MAS, we performed whole-genome sequencing (WGS) in 10 selected MAS cases followed by target gene validation analysis in a larger number of cases. We created a comprehensive genomic map of the molecular aberrations and tumor-specific fingerprints/signatures in MAS at the whole-genome level. We also analyzed the molecular differences in MAS with and without SO, and compared our findings to previously published work.

## Materials and Methods

### Case Selection

The study was approved by the Northwestern University Institutional Review Board (IRB). Two pathologists reviewed the pathology database from Northwestern Memorial Hospital. Among 45 cases with diagnosis of MAS in our database, 29 cases with available research materials and well-documented clinical and follow-up information were included. All tissue samples were taken retrospectively from achieved materials from Northwestern Memorial Hospital of routine hospital care. Ten cases selected for WGS were mainly based on the quantity and quality of tumors, such as recent cases (<6 years), the blocks with large tumor size and sarcoma and epithelia ratio close to 9:1. Another 19 cases of MAS were selected for validation purpose. Patient and disease characteristics including age, surgical procedure, uterine size and weight, tumor size, tumor site, histology, cellular and molecular analyses, stage at presentation, adjuvant therapy, and time to recurrence and/or death were recorded and evaluated (Table 1, Supplementary Table 1, and Supplementary Figure 1). Progression-free survival (PFS) was defined as time from initial diagnosis to recurrence, and overall survival (OS) was defined as time from initial diagnosis to death (Table 1).

**Table 1:** Pathology and clinical information of Müllerian adenosarcoma (MAS).

	MAS for WGS^*^	MAS for TVA^*^
Cases (No.)	10	29^**^
Age (yrs., range)	45.5 (29–86)	44 (22–86)
Tumor size (cm, mean ± sem)	7.5 ± 3.63	5.63 ± 3.40
AS with SO	70% (7/10)	51.7% (15/29)
**AS location**		
Cervix	0 (0/10)	17.2% (5/29)
Uterus	70% (7/10)	69.0% (20/29)
Ovary	30% (3/10)	13.8% (4/29)
**Tumor grade**		
Low grade	40% (4/10)	62.1% (18/29)
High grade	60% (6/10)	37.9% (11/29)
**Tumor stage (FIGO)**		
I	80% (8/10)	89.7% (26/29)
II	0	0
III	20% (2/10)	10.3% (3/29)
IV	0	0
**Clinical outcome**		
ANED	80% (8/10)	86.2% (25/29)
AWD	20% (2/10)	10.3% (3/29)
DOD	0	3.4% (1/29)
Endometriosis	30% (3/10)	20.7% (6/29)

* *SO, sarcomatous overgrowth; TVA, target validation analysis; WGS, whole genomic sequencing analysis; ANED, alive with no disease; AWD, alive with disease; DOD, died of disease; FIGO, International Federation of Gynecology and Obstetrics*.

** *29 cases for target validation include 10 cases with WGS*.

### Genomic DNA Extraction

Twelve consecutive sections (5 μm) of formalin-fixed paraffin-embedded (FFPE) tumor tissue and myometrial tissue were prepared. The first and last sections were hematoxylin and eosin (H&E) stained and reviewed. The remaining 10 unstained sections were used for DNA extraction (containing at least 2–3cm^2^ tumor size) DNA was prepared from tumor sections of 29 cases and from the matched endomyometrial tissue sections of 10 cases. The DNA was extracted and purified using the QIAamp DNA FFPE Tissue Kit (QIAGEN, 56404, Germany), according to the manufacturer's instructions. DNA was quantified using Qubit 2.0 Fluorometer (Life Technologies, Carlsbad, CA, USA) and DNA integrity was checked with 1% agarose gel (Supplementary Figure 2A).

### Library Preparation and Whole Genome Sequencing

NEBNext® Ultra™ II DNA Library Prep Kit for Illumina clustering and sequencing reagents were used according to the manufacturer's recommendations. Briefly, the genomic DNA was fragmented by acoustic shearing with a Covaris S220 instrument. Fragmented DNA was cleaned and end-repaired (Supplementary Figure 2B). Adapters were ligated after adenylation of the 3′ ends followed by enrichment by limited-cycle PCR. DNA libraries were validated using a DNA 1000 Chip on the Agilent 2100 Bioanalyzer (Supplementary Figure 2C, Agilent Technologies, Palo Alto, CA, USA) and quantified using Qubit 2.0 Fluorometer. The DNA libraries were also quantified by real-time PCR (Applied Biosystems, Carlsbad, CA, USA), and multiplexed in equal molar mass. The pooled DNA libraries clustered on 10 lanes. After clustering, the samples were loaded on the Illumina HiSeq instrument according to the manufacturer's instructions. The samples were sequenced using a 2x 150 paired-end (PE) configuration. Image analysis and base calling were conducted by the HiSeq Control Software (HCS) on the HiSeq instrument (Supplementary Figure 2D). WGS was performed with Q30 bases coverage and over 94–95% of Aligned reads (Supplementary Figure 2D, Supplementary Table 2). The genomic data of each of the 10 cases were normalized to a reference genome from *Homo sapiens* (NCBI GRCh38 with decoys, female).

### Bioinformatic Analysis of WGS Data

The WGS data was analyzed using Basepair software (https://www.basepairtech.com/) with a pipeline that included the following tools: reads were aligned to the UCSC genome assembly hg19 using BWA (18) with default parameters, and duplicate reads were removed using Picard Mark Duplicates. Single nucleotide variants (SNV) were discovered using freebayes (19). The variants were annotated and population data was added from dbSNP and gnomAD. Copy number variation (CNV) was detected using GATK, and a panel of normal was created using female samples from Polaris HiSeqX Diversity Panel. Structural variants (large indels, translocations, etc.) were identified using Manta (20).

In brief, data were analyzed as following: ***Alignment:*** Reads resulting from Illumina Hiseq 2500 sequencing were aligned to hg19 (UCSC) and DNA sequences from normal myometrium using BWA (v0.7.5) with parameters “mem –t 8 -P -M” and the generated files were then sorted and PCR duplicates removed using Picard (v1.105) (http://broadinstitute.github.io/picard). Subsequently, the BAM files were indexed by samtools (v0.1.19). ***SNPs and indels calling:*** We detected SNPs and indels according to the GATK4 best practice workflows. After duplicate-read removal using Picard, the BAM files were recalibrated and variants were called using GATK4 with known indels in the 1000 Genomes Project and known SNPs in dbSNP v144. ***Somatic*** ***mutations calling:*** Somatic mutations, including single nucleotide variants (SNVs) and small insertions and deletions (indels), were identified by VarScan (v2.4.1) and further filtered by in-house variant detection software to remove possible false positive mutations. Only somatic mutations with a minimum of 3 variant reads and a variant allele frequency > 0.01 were filtered out for further analysis. ***CNVs detection:*** The Somatic CNVs of sample pairs with WGS were identified by modified method based on Segseq algorithm and copy number gain or loss status using thresholds of Z2.5 copies for gain and r1.5 copies for loss. GISTIC algorithm was used to infer recurrently amplified or deleted genomic regions, using copy numbers in 100-kb windows. G scores represented the frequency and amplitude of amplifications or deletions of each genomic region. ***SV detection:*** After duplicate-read removal using Picard, structural variations were detected for each sample separately using CREST39 with Hg19 as the reference genome. Then, the generated tumor.predSV.txt files were annotated using in-house with Hg19 and dbSNP v144.

### Target Validation for Frequently Mutant Genes Analysis and Structure Variation (SV) In-frame Fusion Gene Analysis

Genomic DNA was extracted as described above. Then the target genes were amplified by multiplex PCR with QIAGEN Multiplex PCR Kit (QIAGEN, 206143) with designed primers (Supplementary Table 3A). PCR products were purified by ExoSAP-IT reagent (Affymetrix, Inc.). A DNA library was prepared using the QIAseq 1-Step Amplicon Library Kit (QIAGEN, 180415). Next generation DNA sequencing (NGS) was performed on the ABI 3730 High-Throughput DNA Sequencer (Genomics Core Facility, Northwestern University) with the read depth of 105,718X (range of 78,090-152,121X). Mutations and variations were analyzed by DNASTAR Lasergene 9 software. Three SV involving in tumorigenic genes were selected and analyzed. Primer pairs to cover the upstream and downstream genes were designed using Primer 6 software (Supplementary Table 3B). Fusion genes/genomic DNA fragments were amplified by PCR with HotStar Taq Master Mix (Qiagen, 203446), and purified by ExoSAP-IT reagent (Affymetrix, Inc.). DNA sequencing was performed on the ABI 3730 High-Throughput DNA Sequencer.

### Tissue Microarrys and IHC

FFPE tissue blocks with MAS were selected for each case and 2 mm tissue cores were taken to create tissue microarrays (TMAs). The TMAs were sectioned at 4 μm intervals consecutively. The first and last slides were H&E stained for quality assurance to confirm the correct tumor type. Based on CNV and pathway analysis, 32 markers were selected for immunohistochemical (IHC) analysis (Supplementary Table 4). After deparaffinization and antigen retrieval, all immunohistochemical staining was performed on a Ventana Nexus automated system (Tucson, Arizona). The percent and intensity of each stain were evaluated by two pathologists independently. The intensity was scored as negative (0), weak (1+), moderate (2+), or strong (3+) and the percentage of positive tumor cells was scored from 0 to 100%. Intensity score multiplied by percentage (H-score) was used as the final semi-quantitative score for each case.

### Statistical Analysis

GraphPad version 6 was used for statistical analysis. Student's *t*-test was use to analyze differences between two groups of continuous data and ANOVA for comparison of three or more groups. *P* < 0.05 were considered to be statistically significant. R-3.0.1 (http://www.r-project.org/) was utilized to create hierarchical clustering and heat maps for the IHC markers by tumor type.

## Results

### Clinical and Pathological Analysis

A total of 29 MAS were selected for this study. The mean age was 44 years (range 22–86 years). The mean tumor size was 5.63 cm (±3.40 cm). Among 29 cases, 5 were from cervix, 20 from uterus, and 4 from ovary. Of note, 20.7% (6/29) of MAS, including 3 from the ovary, had endometriosis. 51.7% (15/29) of MAS contained SO. 62.1% (18/29) were low grade and 37.9% were high grade based on nuclear features and heterologous elements in the sarcoma. All high-grade cases were noted to have SO. The majority of patients had stage I disease (89.7%) while the remaining 10.3% were stage III. The median clinical follow-up time was 57 months (range 2–179 months). Fourteen patients received adjuvant chemotherapy and/or radiation. 86.2% (25/29) were alive with no evidence of disease (ANED), 10% (3/29) were alive with disease (AWD), and one patient had died of disease (DOD). Recurrence was observed in 3 cases. Detailed clinical and pathologic information is summarized in Table 1 and Supplementary Table 1.

### Copy Number Variations (CNVs)

Ten cases, including 7 with SO, were selected for WGS (Table 1). The detailed working steps and remarks were summarized in Supplementary Figure 3. In general, we found an average of 1.5 million SNVs: 50,869 in exons and 21,801 in coding regions. Specifically, nearly 200 insertions and 231 deletions in coding regions were identified (Supplementary Table 2). All SNVs identified might represent the candidates of somatic variants as no matched normal genomic DNA was used in this study.

**Figure 1:**
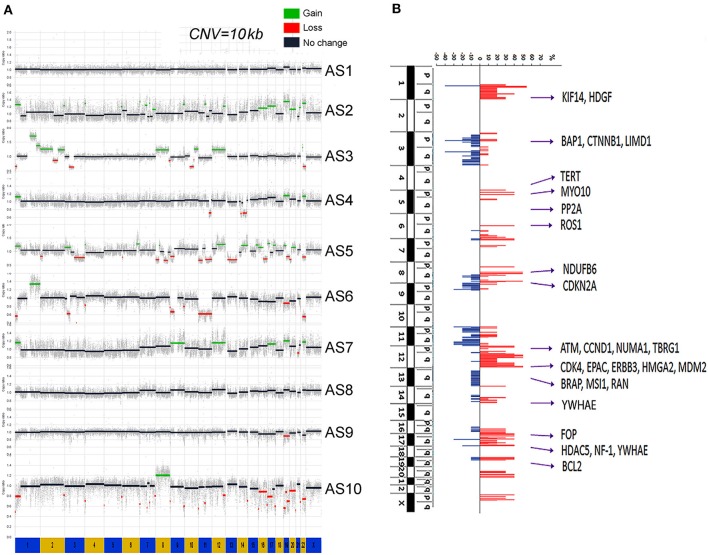
Genome copy number variation (CNV) analysis by whole-genome sequencing (WGS). **(A)** The genomic landscape of copy number gain (green) and loss (red) in each of 10 Müllerian adenosarcomas (MAS, numbered AS-1 through AS-10). **(B)** The accumulated CNV in 10 cases. The scale bars of copy number gain (red) and loss (blue) indicated the percentage of study cases (y axis). A total of 28 genes corresponding to genomic amplification were selected for protein expression analysis.

Copy number gains were relatively common in MAS (Figure 1A). CNV rate per case varied, with an average of 51.3 CNVs per case (range 17–107) (Supplementary Table 2). Gains were found mainly in chromosomes (chr) 1q, 5p, 6q, 7p, 8q, 12p, 12q, and 17q (Figure 1B, Supplementary Table 5). Copy number losses were commonly seen in chr 3p, 3q, 9p, and 11q (Figure 1B, Supplementary Table 5). Large areas of gain or loss were relatively infrequent and mostly found in chr 1q, 3p, 8, and 12q (Figure 1B). The rate of CNVs was much higher in MAS with SO (mean 52.8, range 32–107) than in MAS without SO (45.5, range 17–74). Among the genomic regions with frequent gain or loss (>3 cases), the involved genes were identified (Supplementary Table 5). Further analysis revealed that gains in chr 12q13-q15.1 were present in 6 of 10 MAS (Figure 1B) and involved many genes including *MDM2, CDK4*, and *HMGA2*. In addition, gain in 1q21-q23 was present in 5 MAS, involving *NTRK1, CTSK*, and *HDGF*; gain in 8q13-21 in 5 MAS, involving *ARMC1, MYBL1, PRDM14*, and *TERF1*; and gain in 5p15 in 3 MAS, involving *TERT*. In contrast, copy number loss was relatively infrequent. For example, loss in 3p21 was observed in 4/10 cases (Figure 1B) involving in *BAP1* and 3q13 in 4/10 cases, involving in *LSAMP* and *TUSC7*.

### Immunohistochemistry Analysis of Candidate Genes

**Figure 2:**
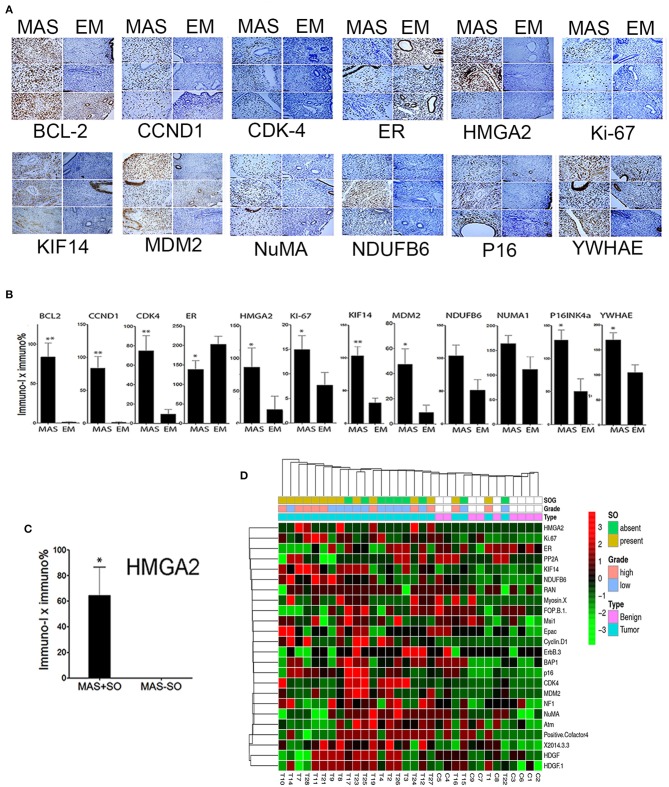
Expression analysis of selected gene products by immunohistochemistry. **(A)** Photomicrographs illustrating the immunohistochemical staining pattern of endometrium (EM) and Müllerian adenosarcoma (MAS) in three selected cases of each. Immunostaining was performed to detect the biomarkers are listed below each panel. **(B)** The relative immunoreactivity (intensity × percentage) of the selected biomarkers is presented in histobars for MAS (*N* = 29) and EM (*N* = 8). Small t bars represent standard error. **(C)** Significant HMGA2 protein expression in MAS with sarcomatous overgrowth (MAS+SO) vs. without (MAS-SO). **(D)** Unsupervised Dendragram Treeviews of protein expression of 24 biomarkers in normal endometrium (benign, blue) and MAS (pink) at different tumor grades and with or without SO (indicated in index bars above) and organ site. All immunoscores from D were normalized to generate the scores from negative to positive for a better visualization of expression pattern. *p < 0.05, **p < 0.01.

We explored candidate gene products according to frequent CNVs. A total of 28 genes were selected for protein analysis, including 11 previously reported to be overexpressed in MAS (Table 2A, Supplementary Table 5, and Figure 1B). The selected gene products were highly relevant to sarcoma carcinogenesis and tumor cell proliferation. Gene products was examined by IHC (Figure 2A) and scored semi-quantitatively (see Methods). Overall, 25 markers showed distinct expression patterns in MAS and tended to be higher in MAS than in normal endometrium, with 40% (10/25) significantly overexpressed in MAS vs. normal endometrium (Figure 2B, Supplementary Table 6). H-score cut-off for positive and negative was calculated in tumor (Supplementary Table 6). Ten selected markers were positive in over 20% of MAS in our study and in published data (Table 2A). Among them, 5 markers (YWHAE, NUMA1, CCND1, KIF14, and BCL2) were first reported in our CNV and IHC analysis (Table 2A). Of note, immunopositivity for YWHAE was found in 68% (17/25) of cases. HMGA2 overexpression significantly correlated with MAS with SO (Figure 2C). MDM2 and NDUFB6 were significantly different among different organ sites (Supplementary Table 6), and CCND1 expression was significantly increased in stage III MAS compared with stage I MAS (*p* = 0.02). There was no significant association between expression of selected biomarkers and recurrence. To evaluate the association of gene expression with tumor grade and SO, an unsupervised cluster analysis was performed. To better present the data and heatmap, the original IHC scores were converted into Z-scores [z-score = (x - μ)/σ; x indicates pre-normalized gene IHC raw score, μ indicates study mean of gene IHC raw scores and σ indicates study standard deviation of gene IHC raw scores). It showed an aggregation of high-grade MAS and SO away from low-grade MAS (Figure 2D).

**Table 2A:** Frequently altered gene products of CNV confirmed by IHC examination in MAS.

		**Howitt et al. (15)**	**Lee et al. (9)**	**Piscuoglio et al. (14)**	**Hodgson et al. (8)**	**This study**	**Total**	**Gain or Loss**
Cases (No.)		18	16	19	18	29	100	
YWHAE^*^	chr17					68% (17/25)	68% (17/25)	Gain
NUMA1^*^	chr11					52% (12/23)	52% (12/23)	Gain
CCND1^*^	chr11					38% (9/24)	38% (9/24)	Gain
KIF14^*^	chr1					32% (8/25)	32% (8/25)	Gain
CDKN2A	chr9	28% (5/18)	19% (3/16)			36% (9/25)	29% (17/59)	Gain
CDK4	chr12	28% (5/18)	31% (6/16)	26% (5/19)	11% (2/18)	33% (8/24)	27% (26/95)	Gain
HMGA2	chr12	28% (5/18)	25% (4/16)	26% (5/19)		24% (6/25)	26% (20/78)	Gain
BCL2^*^	chr18					24% (6/25)	24% (6/25)	Gain
TERT	chr5			21% (4/19)			21% (5/19)	Gain
MDM2	chr12	28% (5/18)	38% (5/16)	26% (5/19)	5% (1/18)	19% (5/25)	22% (21/96)	Gain
RB1	chr13	17% (3/18)	13% (2/16)				15% (5/34)	Loss
MYBL1	chr8	22% (4/18)		5% (1/19)			14% (5/37)	Gain
TP53	chr17		13% (2/16)		11% (2/18)		12% (4/34)	Loss
BAP1	chr3	17% (3/18)		5% (1/19)	5% (1/18)	17% (4/23)	13% (10/78)	Loss
DICER1	chr14				11% (2/18)		11% (2/18)	Gain

* *Newly identified in this study*.

### SV Analysis Indicates High Breakpoints in Chromosome 7

**Figure 3:**
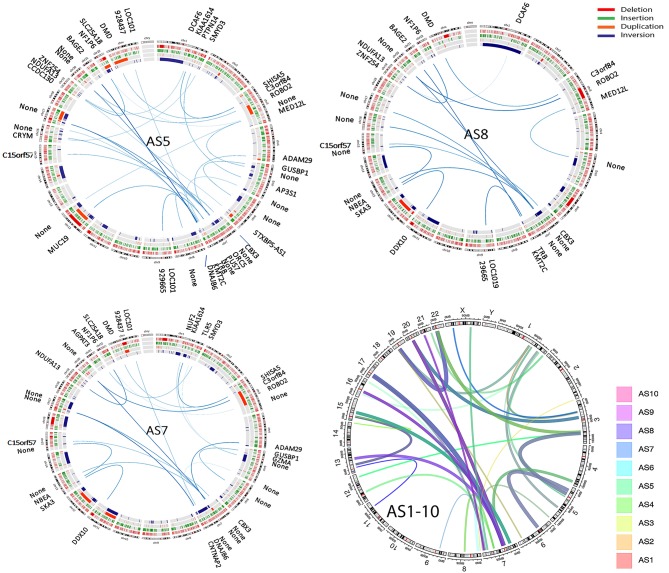
Structure variation (SV) analysis of genomic rearrangements in different chromosomes illustrated by Circos plots. Circos plots of the selected three Müllerian adenosarcomas (AS5, AS8, and AS10) show SVs including deletion (red), insertion (green), duplication (orange), and inversion (blue) detected by next-generation sequencing (NGS) analysis. The lines traversing the ring indicate the genes (denoted outside of the circus plot) or non-gene genomic regions (thickness indicates the relative rate by NGS reads) indicating the in-frame gene or genomic fusion/rearrangement. The final circos plot (right lower) provides a summary of total SV changes from all 10 MAS, with each MAS indicated by the color legend on right of the plot.

**Figure 4:**
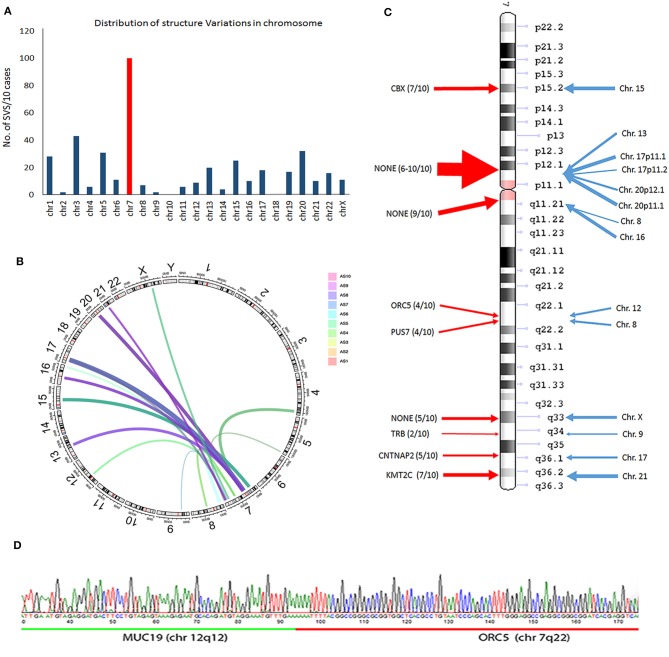
Frequent structure variation (SV) changes in chromosome 7. **(A)** Histoplots show the number of SV changes in each chromosome (chr 7 is in red). **(B)** Circos plot illustrates the inframe gene and genomic rearrangement between chr 7 and other chr partners (assembled from 10 cases; line thickness indicates the relative frequency of SV). **(C)** SV distribution and number of SV changes (thickness of arrows) in chr 7 (red arrows) region and other chr partners (blue arrows). The candidate genes in each SV region are listed. **(D)** An example of SV changes validated by PCR and Sanger sequencing analysis through two different gene fusion regions (upstream gene in green; downstream gene in red).

SVs involve genomic structure changes with or without in-frame gene fusion in different chromosome regions. To better evaluate and visualize the SV, Circos plots were performed for all cases (see Supplementary Figure 5 for detailed information on distribution of deletion, insertion, duplication, and inversion). SV analysis for breakpoint/end change in 10 MAS revealed a relatively low rate of SV events in MAS (Figure 3). There were overall 95 SVs involving 66 genes (Supplementary Table 7). Circos plots showed the identified in-frame gene fusions within or crossing different chromosomes (Figure 3). While most SV changes involved genes (gene names labeled outside of Circos plots), some fusion sites did not contain gene sequences. SV in-frame gene fusions were frequently seen in chr 1, 3, 5, 7, 13, 15, 17, 20, and 22. Interestingly, SV break-ends were almost undetectable in chr 2, 10, 14, and 18 in 10 MAS (Figure 4A).

A frequent SV in-frame gene fusion occurred in chr 7 (Figure 4A, Supplementary Figure 5). There were 15 breakpoints within chr 7, 9 of which were present in 2 or more cases and cross-linked to 12 different chromosomes. Common in-frame rearrangements involved chr 7 and 17 (Figure 4B). The frequent break-ends in 7p12.1 did not contain gene(s) (Figure 4C). The break-ends found in chr 7 were within 6 genes (*CBX3, ORC5, PUS7, TRB, CNTNAP2*, and *KMT2C*), and in-frame gene fusions or non-gene fusion in 12 chromosomes (Figure 4C, Supplementary Table 7). Of note, 7 break-ends in chr 7q were present in at least 4 of 10 MAS, indicating that this is a site of frequent genomic alteration in MAS (Supplementary Table 7). To determine whether WGS-identified SVs were present in tumors, primer pairs of each breakpoint immediately upstream or downstream flanking DNA sequences across different chromosomes were designed, and tumor genomic DNA was generated by PCR for sequencing analysis (see methods and Supplementary Table 3B). Three randomly selected SV breakpoints including 7q22-12q12 (*ORC5*-*MUC19*), 2q14-9q34 (*DPP10*-*SET*), and 12q14-15q15 (*BAZ2A*-*FSIP1*) were analyzed, and all were confirmed by sequencing analysis (Figure 4D).

### Target Validation for Frequently Mutant Genes

**Figure 5:**
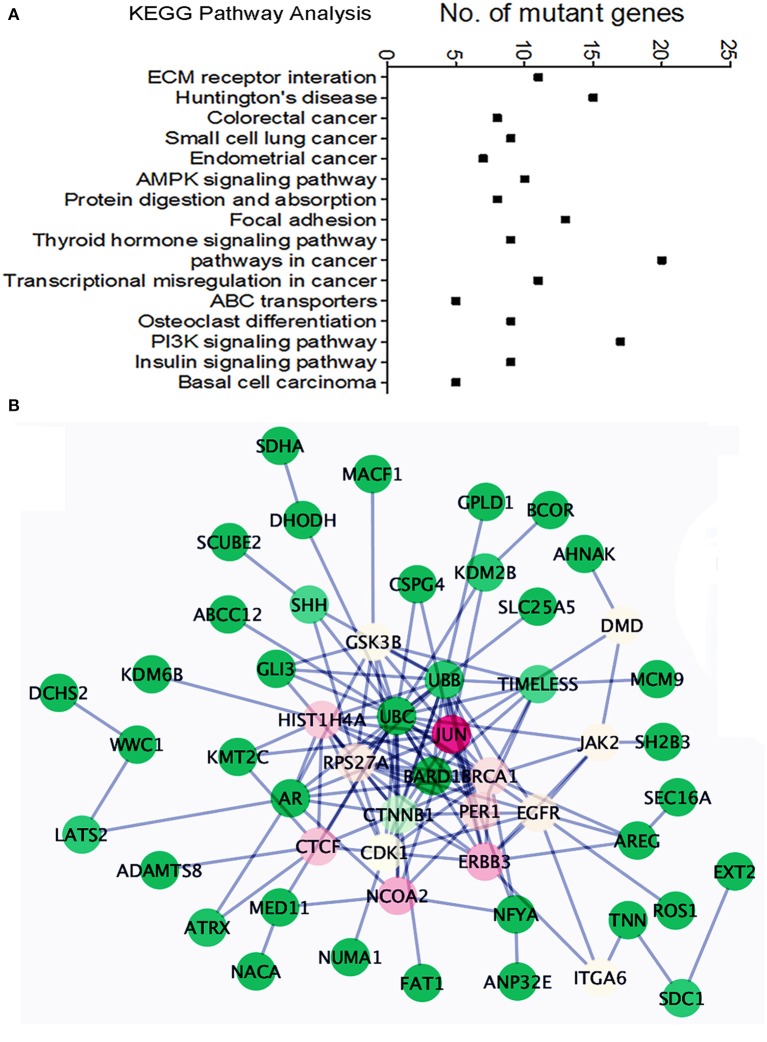
Pathway analysis of the frequently mutated genes in Mullerian adenosarcoma (MAS). **(A)** Dot plot illustrates KEGG pathway analysis of oncogenes/tumor suppressor genes altered in MAS (for details, see text). **(B)** Schematic diagram illustrates the functional connections among 43 mutated genes. The color indicated the value of Betweenness Centrality (green = minimum, red = maximum).

We found an average of 1.5 million single nucleotide variations (SNVs, Supplementary Figure 6): 50,869 in exons, 21,801 in coding regions. Specifically, nearly 200 insertions and 231 deletions in coding regions were identified (Supplementary Table 2). Among 50,869 SNVs in exons, about 310 genes showed one or more missense or nonsense mutations in more than 2 cases. Pathway analysis (KEGG pathway) of these mutant genes indicated dysfunction of many different pathways involving cellular and extracellular functions, *AKT* and *AMPK* signaling, and endometrial cancer-related gene function (Figure 5A). To further detect gene mutations that are frequent in MAS and/or highly relevant to MAS tumorigenesis, we selected candidate genes (defined as hot gene mutations) that met the following criteria for further validation analysis: (1) mutations in at least 2 of 10 cases from WGS; (2) oncogenes or tumor suppressors reported to be closely related to any sarcoma or Müllerian malignancy; (3) altered expression found in MAS.

A total of 40 hot genes were selected for further analysis. We investigated the functional relationships among these 40 mutated genes (Figure 5B) using the Reactome Functional Interaction (FI) plug-in Cytoscape (21). Network analysis based on Betweenness Centrality (a measure reflecting the amount of control one node exerts over interactions of other nodes in the network) highlights nodes that have denser subnetworks. The network graph showed a general overview of all 40 mutated genes and their functional connection with JUN (Figure 5B). Frequently mutated genes in MAS seemed to be centralized by *BRCA1*, by influencing cell cycle function and AKT signaling.

**Figure 6:**
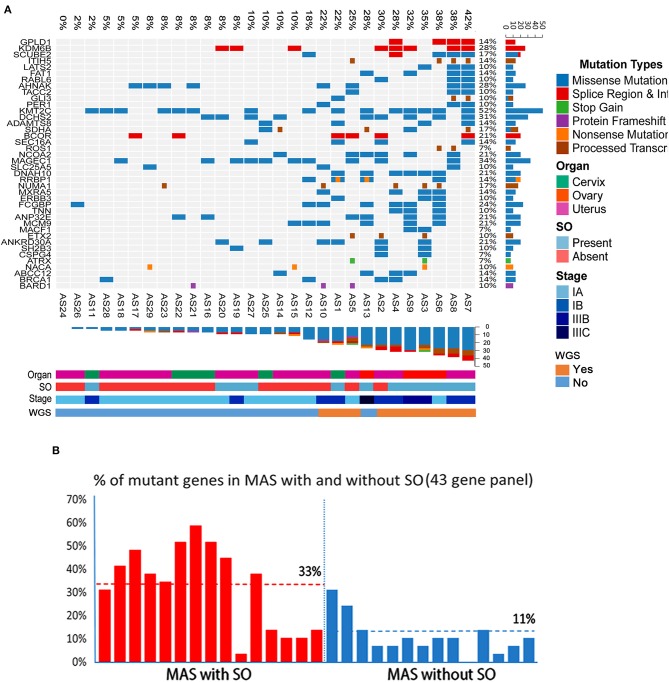
Landscape of frequent mutations of genes in Müllerian adenosarcoma (MAS). **(A)** Target validation of the gene mutation types and distribution of 43 oncogenes/tumor suppressors in 29 MAS by next-generation sequencing (NGS). Rows represent individual genes and columns represent individual tumors. Mutated genes are sorted according to frequency in this cohort. Colors indicate the mutation type detected in each tumor. **(B)** Histoplots illustrate the percentage of 43 mutant genes in MAS with sarcomatous overgrowth (SO, red bars) and without SO (blue bars).

The target genomic DNA sequences from these 40 genes containing the mutation sites were generated by multiplex PCR and analyzed by NGS (Supplementary Table 3A). We first compared the mutation patterns between WGS and NGS target validation in 10 cases and found that 98.2% of mutations were detected by both methods in the same tumors, indicative of a reliable assay for target validation. The mutation frequency (in the range of 7–52%) and mutation types in 40 genes among 29 cases (10 cases from WGS and 19 additional cases) were illustrated in Figure 6A. A high mutation frequency was found in *KMT2C* (52%), *MAGEC1* (34%), *DCHS2* (31%), *KDM6B* and *AHNAK* (28%), and *FCGBP* (24%) (*p* < 0.01). *BCOR* mutations, found 21% (6/29) of cases, were found in splicing sites and did not change amino acids (Figure 6A, Table 2B). We found that MAS with SO had significantly higher number of mutant genes (33%) that those without SO (11%) (Figure 6B). Together, 9 frequently mutant genes had not been previously reported (Table 2B). Eight other mutant genes reported previously showed similar mutation frequency among our study and previous studies (Table 2B).

**Table 2B:** Frequently mutated genes in MAS.

		**Howitt et al. (15)**	**Piscuoglio et al. (14)**	**This study**	**Total**	**Protein coding changes**
Cases (No.)		18	19	29	66	
KMT2C	chr7		11% (2/19)	52% (15/29)	35% (17/48)	p391(Cys < ns), p384 (Asp < Asn), p380 (Arg < Leu), p309 (Pro < Ser)
MAGEC1^*^	chrX			34% (10/29)	34% (10/29)	p176 (Val < Leu), p178 (Ile < Leu), p232 (Pro < Ser), p260 (Phe < Ser), p267 (Ser < Pro), p292 (Gln < His), p302 (Pro < Leu), p447 (Val < Gly)
DCHS2^*^	chr4			31% (9/29)	31% (9/29)	p2824 (Pro < Ala), p2820 (Val < fs), p1602 (Thr < Ala), p1438 (Arg < Leu)
AHNAK^*^	chr11			28% (8/29)	28% (8/29)	p3378 (Ile < Thr), p2182 (Thr < Ala)
KDM6B^*^	chr17			28% (8/29)	28% (8/29)	p221 (Glu < Asp), p308 (Ser < Leu), p340 (Arg < Pro), p444 (Ser < Gly), p482 (Pro < Ser), p511 (Pro < His), p968 (Val < Gly), p1643 (Arg < Cys)
FCGBP^*^	chr19			24% (7/29)	24% (729)	p4950 (Arg < Gln), p2640 (Glu < Lys), p2433 (His < Tyr), p1343 (Ala < Val), p1333 (Gly < Arg), p645 (Gly < Glu)
ANP32E^*^	chr1			21% (6/29)	21% (6/29)	p47 (Gly < fs), p46 (Tyr < fs), p164 (Glu < Glu)
MCM9^*^	chr6			21% (6/29)	21% (6/29)	p1093 (Met < Val), p898 (Ser < Phe), p816 (Glu < Asp)
NCOA2^*^	chr8			21% (6/29)	21% (6/29)	p1363 (Gly < Arg), p653 (Glu < Val), p407 (Ala < Ser)
ANKRD30A^*^	chr10			21% (6/29)	21% (6/29)	p274 (Ser < Pro), p276 (Val < Ala), p426 (Cys < Trp)
BCOR	chrX	22% (4/18)		21% (6/29)	21% (10/47)	c.4977-4G>T splice site
DNAH10	chr12		5% (1/19)	21% (6/29)	16% (7/48)	p1186 (Glu < Lys), p1489 (Met1 < ns), p1896 (Val < Met), p2735 (Gln < His), p3115 (Lys < Glu), p3620 (Thr < Ile)
GLI3	chr7	11% (2/18)		10% (3/29)	11% (5/47)	p1028 (Ser < Ile), p727 (Gly < Arg), p45 (Ser < Arg)
EXT2	chr11	11% (2/18)		10% (3/29)	11% (5/47)	p161 (Arg < Trp), p369 (Val < Met), p426 (Arg < Gln
ROS1	chr6	17% (3/18)		7% (2/29)	11% (5/47)	p1054 (Ser < Arg), p894 (Arg < Trp), p827 (Trp < Leu)
SEC16A	chr9		5% (1/19)	14% (4/29)	10% (5/48)	p641 (Arg < Cys), p346 (Arg < His)
ATRX	chrX	17% (3/18)	5% (1/19)	7% (2/29)	10% (6/66)	p1752 (Gln < Pro), p865 (His < Gln)

* *Newly identified in this study*.

## Discussion

In this study, we employed a comprehensive approach by conducting a WGS analysis in a cohort of 10 MAS followed by a validation analysis in additional 19 cases. We further compared our results with those of published studies, providing a broader scope of the genetic alterations specific to this disease. Overall, we found that MAS demonstrates some similar CNV patterns among the published studies: a copy number gain in chromosome 12q (8, 9, 14–16). In this study, we found that 7 out of 10 MAS gained in chromosome 12q13-q15, involving target genes *MDM2, CDK4, HMGA2, CDK2, STAT2*, and *STAT6*. Further expression analysis by immunohistochemistry in 29 cases of MAS confirmed overexpression of the selected genes in this region. Gain of chromosome 12q is a characteristic genomic alteration or landmark in most MAS, and HMGA2 gain/overexpression is significantly associated with MAS with SO and seems to be associated with tumor progression.

Other reported findings include gains in *TERT, STAT6, SGK1*, and *DICER1* and losses in *CDKN2A, BAP1, RB1, NF1*, and *TP53* (9, 15). Abnormalities in the PIK3-AKT-PTEN pathway, including CNVs and mutations, are also common (26–72% of cases) (8, 14, 15). MAS with SO demonstrated a higher rate of CNVs, and more losses than gains, compared to MAS without SO (9, 14, 15). This is also consistent with our data, which demonstrated frequent gains in chr 1q, 5p, 6q, 7p, 8q, 12p, 12q, and 17q. Specifically, copy number losses were less frequently seen, and most often involved chr 3p, 3q, 4q, 9p, and 11q. The rate of CNVs (especially loss) was much higher in MAS with SO than in MA without SO (Supplementary Figure 4).

The mesenchymal component in MAS morphologically may resemble low-grade endometrial stromal sarcoma (LGESS). The characteristic genetic alteration in LGESS is seen in the fusion gene *JAZF1/SUZ12* [*t*_(7;17)_] (22). A similarly specific genetic alteration is apparently lacking in MAS. If MAS develops from endometrial stromal cells, further exploration of possible genetic alterations may greatly facilitate our understanding of MAS. Piscuoglio et al. reported two cases with fusion genes involving NCOA family members (14). Traditionally, in-frame gene fusions in many malignancies, including LGESS (22), were detected by cytogenetic analysis and confirmed by positional cloning of the fusion gene products. Using conventional cytogenetics, Howitt and colleagues found chromosomal abnormalities in 43% of cases; 71% of the 7 cases with noncomplex chromosomal aberrations demonstrated abnormalities in chr 8, which contains *MYBL1* (16). Blom and Guerrieri found all MAS with SO and a subset without SO to have aneuploidy (17). The detection of SV and in-frame gene fusions by WGS is a powerful approach, allowing the exploration of SV in neoplasms that may harbor in-frame gene fusions. We noted that while overall SV is relatively low in MAS, it is disproportionately enriched in chr 7. Among 9 break-ends in chr 7, 6 had in-frame fusion genes. A pilot test of PCR through the several break-ends confirmed specific in-frame gene fusions. Further analysis of these gene fusions may provide insight into the genetic mechanisms relevant to MAS tumorigenesis.

The global mutational landscape of MAS is of great interest and remains unknown. Previous studies using oncogene panels and exon sequencing highlighted some hot gene mutations. Howitt *et al* reported that the mean number of mutations in MAS with SO (mean 9.7; range 3–14) did not differ significantly from that in MA without SO (mean 9.6; range 5–16). In this study, we found a significantly higher number of mutated genes in MAS with SO (Figure 6B). Of note, ovarian MAS seemed to have higher number of mutated genes (Figure 6A) and higher expression of the selected gene products (Supplementary Table 6) than cervical and uterine MAS. This may be related to ovarian MAS with higher rate of high-grade and advanced stage of diseases (Supplementary Table 1). Further analysis of MAS from different anatomic sites requires a large number of cases in the future.

Notably, *TP53* mutations were uncommon, present in only two cases with SO. Three out of 18 cases (17%) had mutations in *ATRX*, all associated with SO (15). Piscuoglio et al. reported that only three genes, *FGFR2, KMT2C*, and *DICER1*, were recurrently mutated, all in 2/19 cases (14). Based on WGS analysis in 10 MAS, we found a total of 310 genes with missense or nonsense mutations. These genes involve 16 functional pathways based on KEGG analysis, specifically in cancer, ECM, and AKT signaling (Figure 5A). We performed target validation on 43 selected oncogenes/tumor suppressors and BRCA1 is at its center functionally connected to other mutated genes (Figure 5B). Findings of increased alterations of BRCA1, MDM2, and TP53 in MAS may partially explain a high DNA instability and frequent CNVs in this tumor type, in particular in SO.

This study also broadens our knowledge of the mutation spectrum in MAS and identified many new mutations, including in *KMT2C* (52%), *MAGEC1* (34%), *DCHS2* (31%), *KDM6B* and *AHNAK* (28%), *FCGBP* (24%), and *BCOR* (21%). We reviewed and compared the mutated genes identified in this study with cBioportal and Cosmic datasets. All these mutated genes showed a wide range of alteration in some cancers or sarcomas. In particular, 17 of them were recorded as cancer genes in OncoKB Caner Gene List. The prevalence of mutations of these genes in endometrial cancer and other cancer in cBioportal are 9.5% (1.5–19.6%) and 3.1% (0.5–8.4%), respectively. Apparently the frequently mutated genes in Mullerian adenosarcoma seem to be closely relevant to endometrial cancer.

*KMT2C* mutation in MAS was reported in a previous study (14). *KMT2C* (lysine-specific methyltransferase 2C) is a putative tumor suppressor that is frequently mutated in many malignancies (23) and associated with tumor aggressiveness (24). In particular, KMT2C-mediated ER signaling is critical for ER-positive breast cancer (25). Current studies indicate that *KMT2C* mutation may cause loss of function in MAS and other malignancies (26). The high rate of *KMT2C* mutation and its high rate of SV in chr 7 suggests an important role in MAS. This study also identified a relatively high rate of *BCOR* mutations in 21% MAS. *BCOR* mutation was found in 4 MAS with SO and in 2 MAS without SO. The role of *BCOR* mutation in MAS tumorigenesis merits further investigation.

A few studies examining the immunohistochemical features of adenosarcoma have found that the stromal component stains similarly to LGESS (positive for ER, PR, WT-1, and CD10), and that SO is associated with a decrease in ER, PR, and CD10 expression as well as a higher Ki-67 proliferation index and increased expression of p53 (6, 17, 27–31). However, specific immunohistochemistry markers for the diagnosis of MAS are lacking. To search for potential biomarkers specific to MAS, we examined a group of 24 oncogenes or tumor related gene products detected by CNV analysis, and found that most of them, including BCL-2, CDKN2A, YWHAE, CCND1, HMGA2, KIF14, CDK4, and MDM2, were significantly overexpressed in MAS. HMGA2 was the only biomarker that was significantly overexpressed in MAS with SO. Thus, HMGA2 may be useful in evaluating the tumor nature in biopsy specimens. We did note that many other mutant genes are different in MAS with or without SO. For example, while *KMT2C* mutations can be found in both types, mutations of *KDM6B* and *MAGEC1* seem to be relatively specific for MAS with SO.

In summary, we used WGS in conjunction with molecular target gene analysis to reveal the landscape of genomic alterations/gene mutations in MAS. Our results show a wide spectrum of genetic changes, with complex and relatively disease-specific patterns. In particular, we identified CNV in chr 12q leading to HMGA2/CDK4 upregulation, a high rate of SV in chr 7q leading to several in-frame gene fusions, a high rate of *KMT2C* mutation, and gene mutation signatures specifically related to MAS tumor progression. These findings provide us with a molecular fingerprint of MAS and indicate the potential role of genetic changes in tumor development and tumor prognosis. Additional studies will further aid in defining the specificity of genetic alterations in this tumor type and uncovering genetic mechanisms in MAS and other Müllerian stromal neoplasia.

## Data Availability Statement

The original contributions presented in the study are publicly available. This data can be found here: NCBI SRA (https://www.ncbi.nlm.nih.gov/sra/) (Accession: PRJNA612343).

## Author Contributions

JJ-W and SB designed study. YB, JF, and YL conceived and carried out experiments. YB, JF, HG, CZ, QZ, XW, WZ, KM, SB, and J-JW analyzed data. J-JW, YB, SB, and KM were involved in writing the paper and had final approval of the submitted and published versions.

### Conflict of Interest

The authors declare that the research was conducted in the absence of any commercial or financial relationships that could be construed as a potential conflict of interest.
