# Supplementary material for: Whole-Genome Sequencing and Target Validation Analysis of Müllerian Adenosarcoma: A Tumor With Complex but Specific Genetic Alterations
Source: Front Oncol. 2020 Apr 15;10:538. doi: 10.3389/fonc.2020.00538 (PMC7174971; doi:10.3389/fonc.2020.00538)
Supplement: Supplementary file 1 [file Data_Sheet_1.docx]

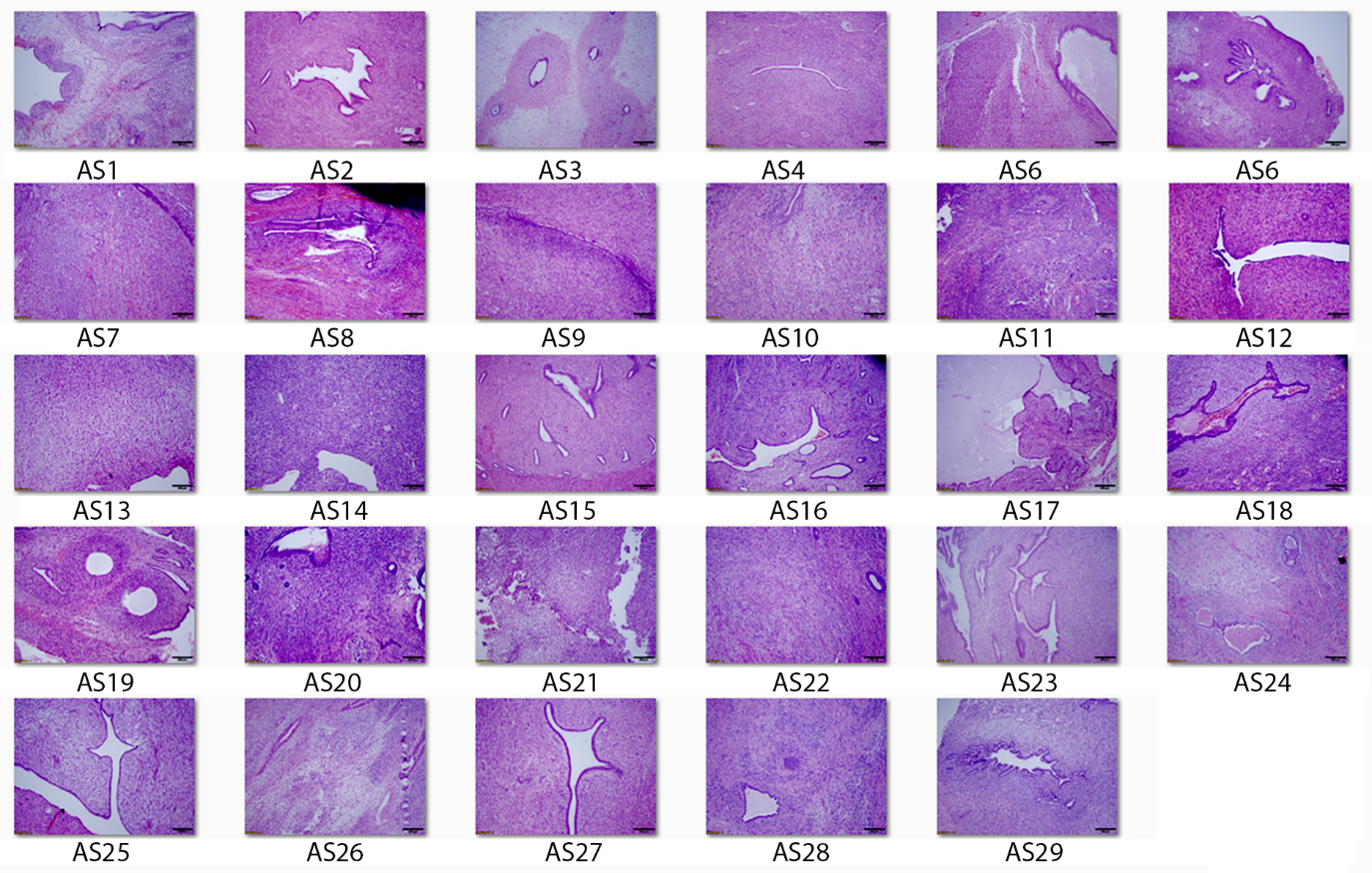


**Suppl Figure 1**Photomicrographs illustrate the histology of 29 cases selected for this study.


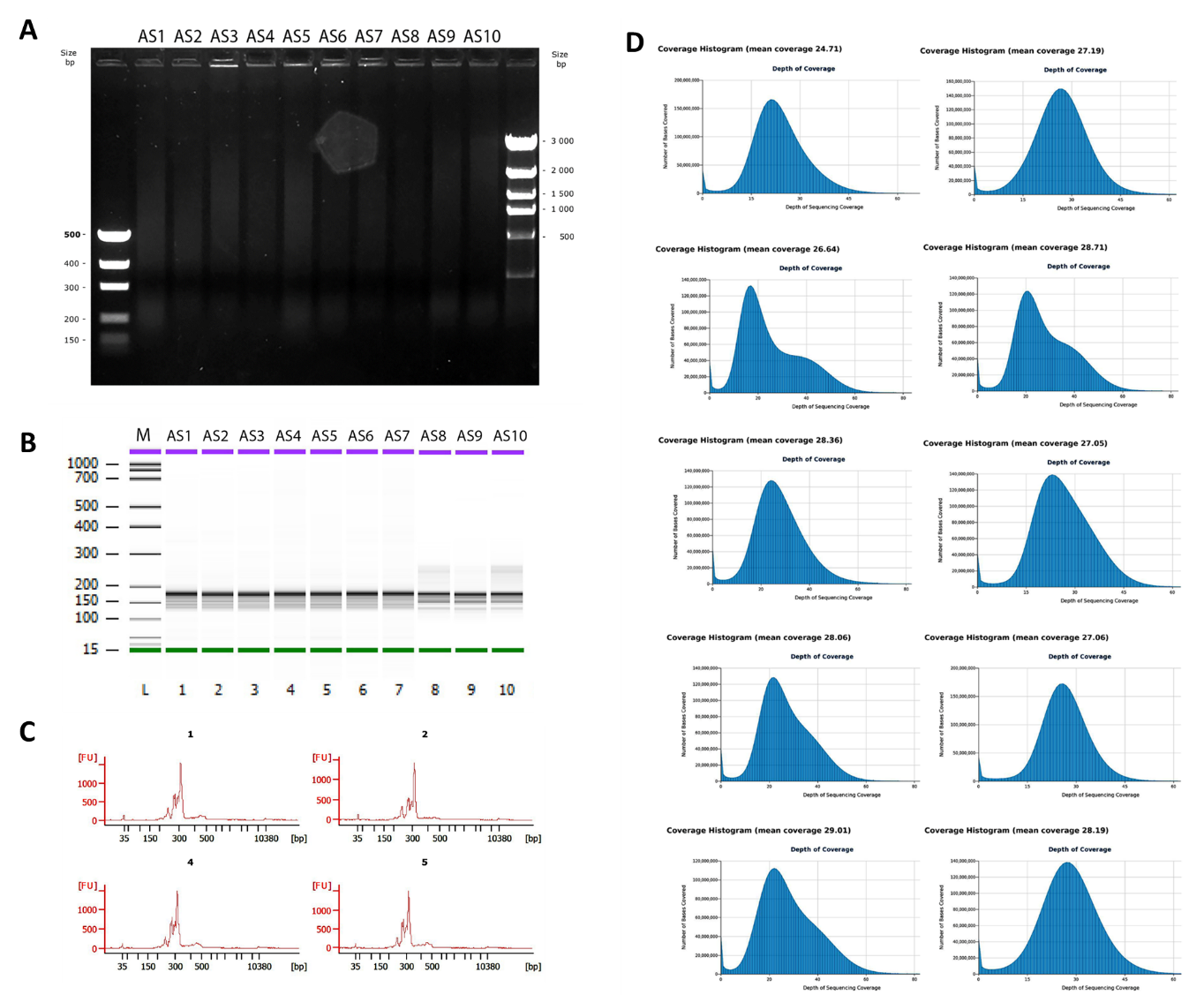
s. **Suppl Figure 2** DNA preparation and quality analysis for whole genome sequencing analysis. A. DNA quality and size from 10 cases of formalin fixed and paraffin embedded tissue tumor sections detected by electrophoresis. B and C. DNA quality analysis from DNA library preparation detected by electrophoresis and plot analysis. D. Depth of sequencing coverage by NGS from 10 case
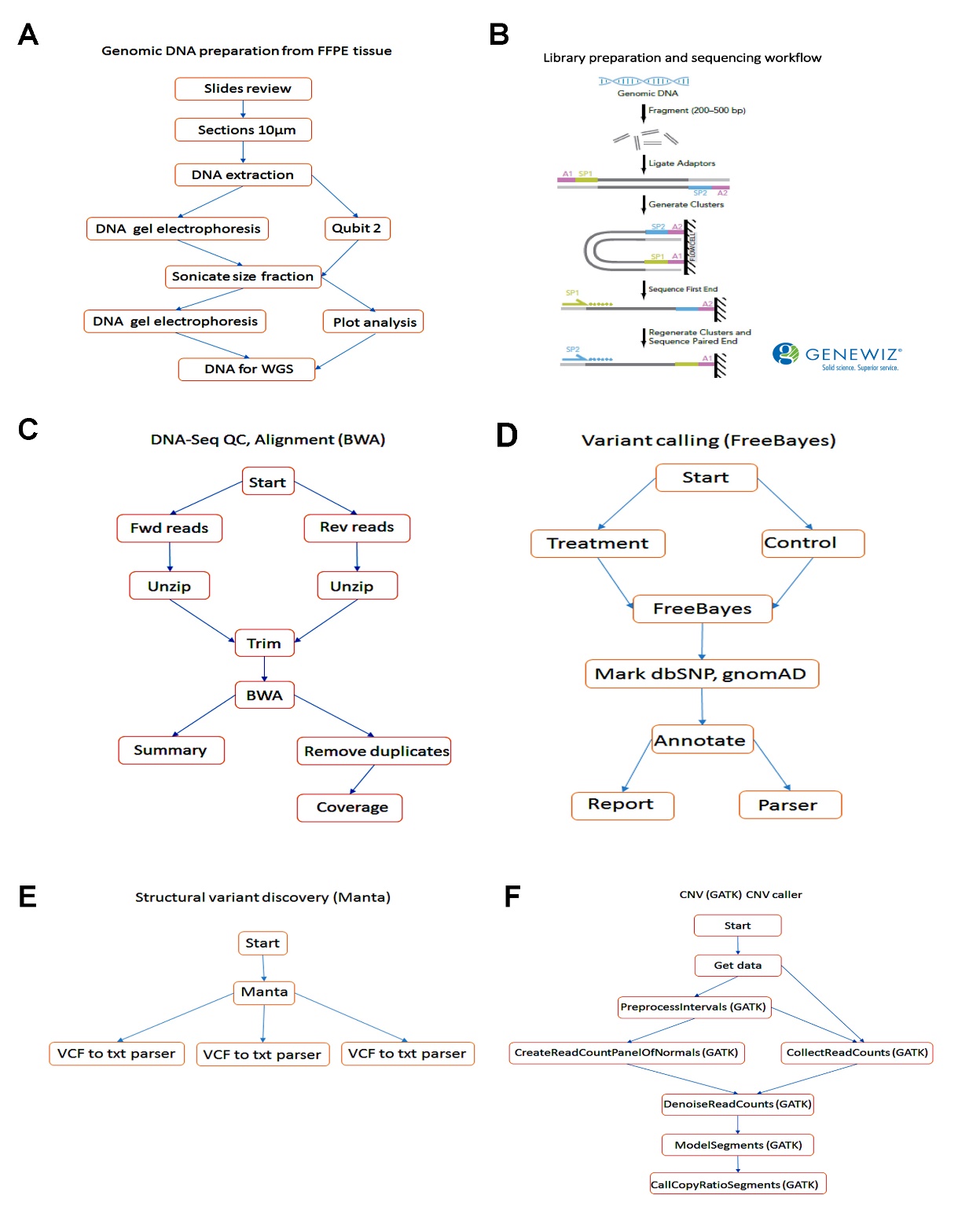


**Suppl Figure 3** Remarks and sketch diagrams illustrating the DNA preparation (A), DNA library preparation (B), WGS DNA quality control steps (C), and genomic DNA sequencing analysis by variation calling (D), SV (E) and CNV (F).


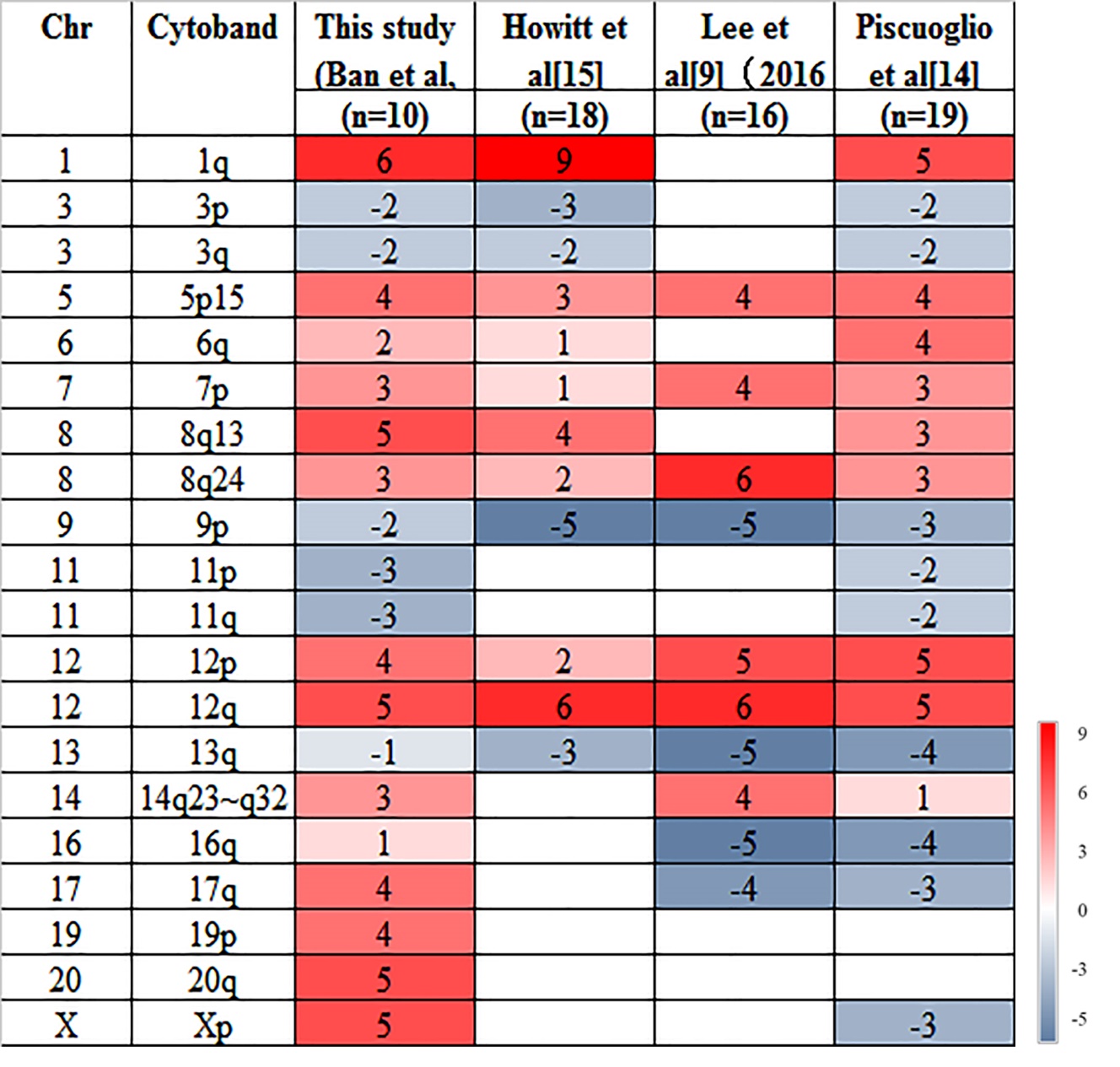


**Suppl Figure 4** Comparison of the common genetic alterations identified in this study with three published data. A. Copy number variation of gain (red) and loss (blue) in genomic and chromosome regions are indicated. The number of cases and level of CNV are shown.

**
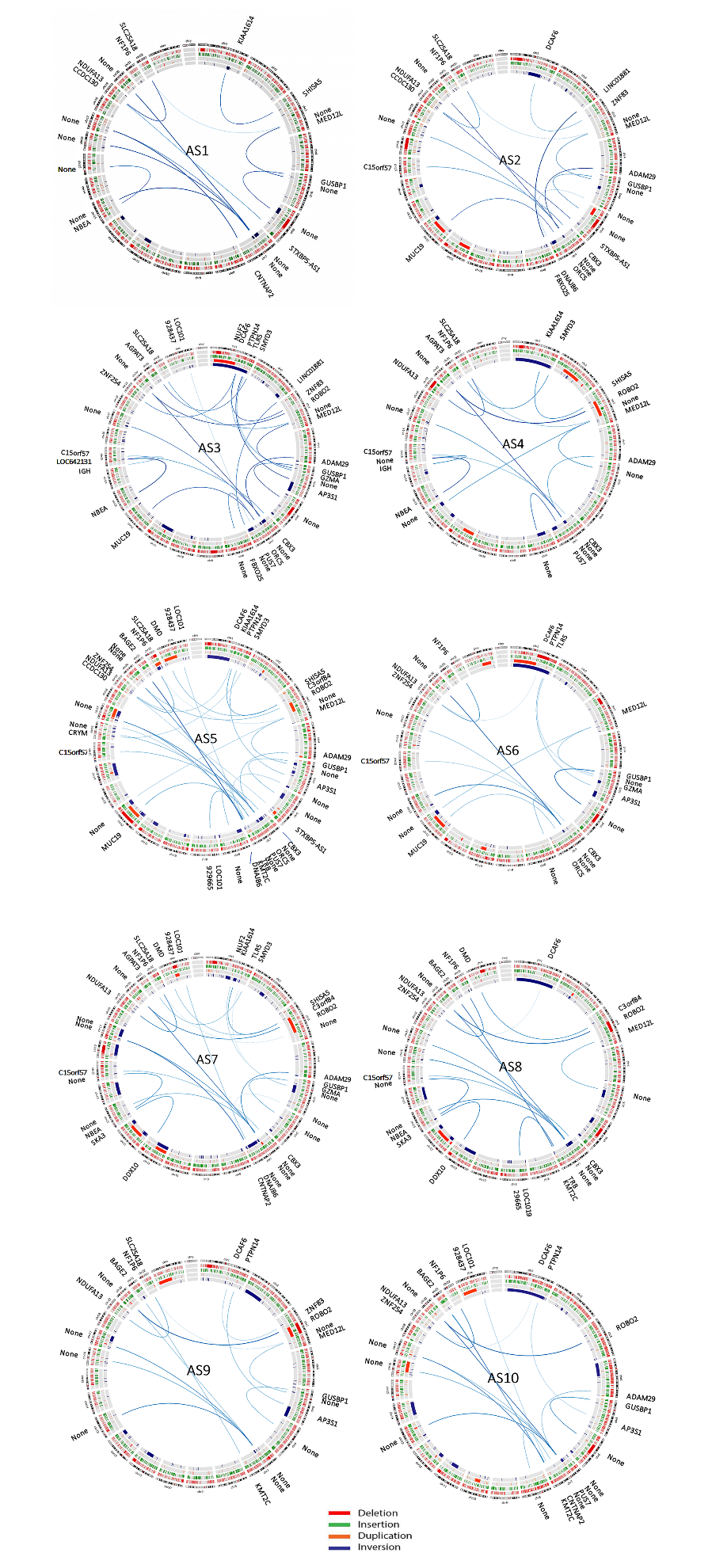
Suppl Figure 5.** Circos plot analysis of genomic deletion (red), insertion (green), duplication (orange), and inversion (blue) in each of 10 (AS1 through AS10) Müllerian adenosarcomas. The lines traversing the ring indicate the genes (denoted outside of the circus plot) or non-gene genomic regions (thickness indicates the relative rate by NGS reads) indicating the in-frame gene or genomic fusion/rearrangement.


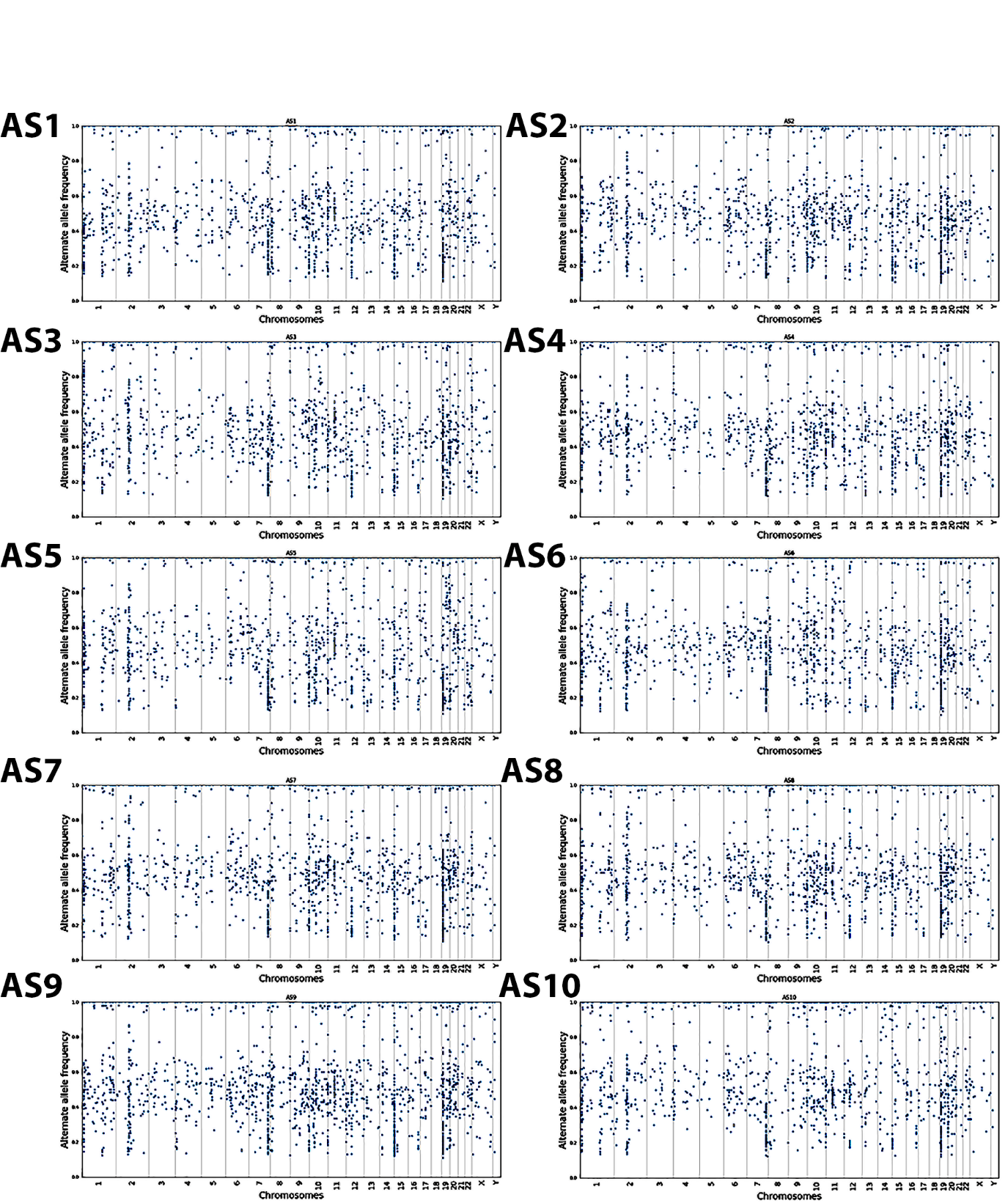


**S Figure 6** Plot analysis of SNP (single nucleotide polymorphism) distribution in chromosome regions in 10 Müllerian adenosarcoma.
