# Supplementary material for: Whole-Genome Sequencing and Target Validation Analysis of Müllerian Adenosarcoma: A Tumor With Complex but Specific Genetic Alterations
Source: Front Oncol. 2020 Apr 15;10:538. doi: 10.3389/fonc.2020.00538 (PMC7174971; doi:10.3389/fonc.2020.00538)
Supplement: Supplementary file 2 [file Data_Sheet_2.docx]

**Suppl Table 1 General information of the Müllerian adenosarcoma included in this study**

Case Age Size Procedure Site Grade SO Stage Recurrent WGS ENMS

AS01 29 3.3 TAH Cervix high Y IB y

AS02 45 6.2 TAH Uterus Low IB Y

AS03 86 15 BSO Ovary low y IIIB y y Y

AS04 48 5.0 TAH+BSO Uterus low y IB y

AS05 80 7.0 TAH+BSO Uterus low IA y

AS06 41 8.5 TAH+BSO Ovary high y IA y Y

AS07 43 6.0 TAH+BSO Uterus high y IB y

AS08 43 6.0 TAH+BSO Uterus high y IB y

AS09 49 4.0 TAH+BSO Ovary low y IIIB y y Y

AS10 41 12.0 TAH+BSO Uterus high IB y

AS11 22 5.5 TAH Cervix high y IB

AS12 44 7.0 TAH+BSO Uterus high y IA

AS13 38 9.6 TAH+BSO Ovary low y IIIC

AS14 34 3.0 TAH+BSO Uterus low IA

AS15 50 2.0 TAH+RSO Uterus low IA Y

AS16 51 0.7 TAH+BSO Cervix low IA

AS17 25 4.9 TAH Uterus high y IA Y

AS18 55 4.5 TAH+BSO Uterus high Y IA

AS19 41 5.9 TAH+BSO Uterus low y IB

AS20 69 11.4 TAH+BSO Uterus high y IA Y

AS21 41 3.0 TAH+BSO Cervix low IA Y

AS22 63 2.4 TAH+BSO Cervix low IA

AS23 72 2.5 TAH+BSO Uterus low IA

AS24 40 2.2 TAH+BSO Uterus low IA

AS25 41 3.2 TAH+BSO Cervix low IA

AS26 58 3.0 TAH+BSO Uterus low IA

AS27 68 7.0 EMC Uterus low Y IA

AS28 58 3.0 TAH+BSO Uterus low IA

AS29 44 5.2 TAH+BSO Uterus low IA

* TAH: Total hysterectomy; BSO: bilateral salpingo-oophorectomy; SO: sarcomatous overgrowth; WGS: whole genomic sequencing. ENMS: endometriosis.

**Supplementary Table 2 The data summary for the sequence quality in ten cases of Müllerian adenosarcoma**

Sample ID AS1-10 Mean

READS Total PF Reads 745,609,677.80

Total Aligned Reads-1 355,256,601.60

Total Aligned Reads-2 350,251,382.60

DEPTH OF COVERAGE Percent Q30 Bases 90%

READ LEVEL STATSITICS Percent Aligned Reads-1 95%

Percent Aligned Reads-2 94%

SNVs In Genes 1513193.20

In Exons 50869.20

In Coding Regions 21801.20

In UTR Regions 29068.00

In Splice Site Regions 2655.30

INSERTIONS In Genes 202765.70

In Exons 4164.20

In Coding Regions 199.20

In UTR Regions 3965.00

In Splice Site Regions 284.10

DELETIONS In Genes 209037.00

In Exons 4268.90

In Coding Regions 231.70

In UTR Regions 4037.20

In Splice Site Regions 324.90

STRUCTURE VARIANTS

TOTAL VARIANT TYPES CNVs 51.30

SV Insertions 1553.00

SV Deletions 3608.00

SV Tandem Duplications 74.50

SV Inversions 144.70

SV Breakends 398.40

VARIANT TYPES

CNVs 37.00

SV Insertions 702.40

SV Deletions 1655.00

SV Tandem Duplications 33.90

SV Inversions 86.20

SV Breakends 124.90

**Suppl Table 3A Primer sequence information for target validation of gene mutations**

| **Name** | **Exon** | **Amino acid change** | **5'-3' forward sequence** | **5'-3' reverse sequence** | **Product size (bp)** |
| --- | --- | --- | --- | --- | --- |
| ABCC12 | 24 | p.Pro1134His | CGACTGTCTGCCCACTTTGTAT | CTGTGTTCCTGAATGCACTCATC | 164 |
| ABCC12 | 22 | p.Thr1037Ala | GGCATTACCTGGATGATGTATGACA | AAAACTCCAGTCACCTCCTCTACT | 175 |
| ABCC12 | 21 | p.Ile1000Val | GGTGATGCAGCTCTCCTTCTTG | CAATTTTCTTCCAGCATTTTCCACAGA | 150 |
| ABCC12 | 21 | p.Leu915Phe | GGTGATGCAGCTCTCCTTCTTG | CAATTTTCTTCCAGCATTTTCCACAGA | 150 |
| ADAMTS8 | 7 | p.Phe638Leu | GGATCTGAGTCCCAGGGTGATT | AATTACACTGACATGGACGGGAAT | 156 |
| ADAMTS8 | 7 | p.Gly605Arg | CCTTGGCCTCGAACACTTTGAA | GCAGTGTGAGAAGTATAATGCCTACAATTA | 155 |
| ADAMTS8 | 5 | p.Arg442Cys | CGTCCTGAGCAGAGGTGTT | TCCTCTTTTCCCAGGAGACTGT | 161 |
| ADAMTS8 | 1 | p.Gly93Val | ATGGTGAACTCCTCGCCG | TCAAGATCGAGCGCCTCG | 166 |
| AHNAK | 5 | p.Ile3378Thr | CTTGGATCCTTTCACTTTTCCTTGG | AAAGAAATCTCGTTTTAAGCTTCCCAAATT | 166 |
| AHNAK | 5 | p.Thr2181Ala | GGGCCTTTCAAGTTTAAGTTCACA | TTTGCCAAAATTGGAGGGAGATTTAAC | 174 |
| ANKRD30A | 7 | p.Ser274Pro | GTGGAAAGAACACCTGACACG | TGACTGTTCGAACTTTCCAGATGT | 132 |
| ANKRD30A | 8 | p.Cys426Trp | CTTTTTATAGATCAGAGGTTCCCATCAGAA | GCCTCTGACTTTATATTTTCACATCACTCA | 144 |
| ANP32E | 7 | p.Gly47fs | CCCTTCTTCAACATAGTCATCATCATCT | AGGTTGTCAAAACACAGAATAACCATAGT | 153 |
| ANP32E | 7 | p.Tyr46fs | CCCTTCTTCAACATAGTCATCATCATCT | AGGTTGTCAAAACACAGAATAACCATAGT | 153 |
| ANP32E | 5 | p.Glu164= | CAGCAGAGGCTATAATTATGACACACT | GTGAGATCACAAACCTGGAAGATTATAGAG | 175 |
| ANP32E | 5 | p.Gln92= | CAGCAGAGGCTATAATTATGACACACT | GTGAGATCACAAACCTGGAAGATTATAGAG | 175 |
| ATRX | 26 | p.Arg2028* | ATCTTTAGGAAGGAAGGAAAAGCAACA | AGCATTCTGGGAAAATGGTACTTCTC | 175 |
| ATRX | 20 | p.Gln1752Pro | ATCTGAGGAAATACCAATATTCTACTGCATA | CTGCTGTTTCTAAAGCTATGAATTCTATACGA | 137 |
| ATRX | 9 | p.His865Gln | GAAGAGAAAGTCTCTCTCTCTTGTTTTCT | GTGCTGCCAGAACCACCAAAAA | 175 |
| BARD1 | 11 | p.Ile738Val | GACATCACACAGTCTATAAACCAGCT | GTGACGTGACTCAGACCATCAA | 175 |
| BARD1 | 10 | p.Ser660Gly | AATCAGTCACCTGTAGCTGTTGAAA | GTGAACAGGAAGAAAAGTATGAAATTCCTG | 172 |
| BARD1 | 4 | p.Leu359_Pro365del | GAAGGTGGTGTACCTGGTGAAA | CATTCTGAGCACCAGTGGAGAT | 174 |
| BCOR | Intron14-15 | c.4977-4G>T | ACGTTTGGAAAATTGCAGCGAA | TGGTCCACTTGGGTTGGTAGTTA | 174 |
| BRCA1 | 15 | p.Met1673Ile | ATTAGGGAGATACATATGGATACACTCACA | GAAGAAAGTGTGAGCAGGGAGAA | 131 |
| BRCA1 | 10 | p.Gln356Arg | TGAATGCTGCTATTTAGTGTTATCCAAGG | TGGAAGTAAGGAAACATGTAATGATAGGC | 174 |
| CSPG4 | 10 | p.Pro2294Leu | CACCCAGTACTGGCCATTCTT | TGGTGACACCGAGACCTTTC | 160 |
| CSPG4 | 3 | p.Ala863Val | AGAAATATGGTGGAGCTGTGACAC | TTCAACTACAGGGCACAAGGCT | 150 |
| DCHS2 | 25 | p.Pro2824Ala | GCATTCTTGGTGGGACTGCTTT | CAAAACTAAAGGATGAACATTTGCATATGC | 126 |
| DCHS2 | 25 | p.Val2820fs | GCATTCTTGGTGGGACTGCTTT | CAAAACTAAAGGATGAACATTTGCATATGC | 126 |
| DCHS2 | 18 | p.Thr1602Ala | AAACATTGACTGGGTCTCTGCAA | CCAGTTTTGGAACAGAACCCTTTTG | 147 |
| DCHS2 | 18 | p.Arg1438Leu | ACTGTGGTCATTGGCATCCAAA | GAGTGCTTTACTATAAATGAGATGTCAGGA | 171 |
| DNAH10 | 22 | p.Glu1186Lys | TTCACTTCCTGAGTAAAGACATTGAAAAAC | TTTATCAAGATCATCACCAACAGAACCA | 170 |
| DNAH10 | 27 | p.Met1518Val | CCTGTTCTGGGCTCAAGAACAG | CTGACGTTCTGTAGGTCACTGA | 160 |
| DNAH10 | 33 | p.Val1896Met | CCCTTGTCCTGACATGTCTTTCT | GTGGTTAACTGATGGATCAGAGCA | 175 |
| DNAH10 | 49 | p.Gln2735His | GTCCTTAAGTGGGCTTCTGTTTCA | GCCTCGTAGTCCTGGATGTCTT | 175 |
| DNAH10 | 56 | p.Lys3115Glu | CAGAGGAAAAGGCCATGGAGATA | ACTTGTCCAGCTTCTGCAGTTC | 135 |
| DNAH10 | 63 | p.Thr3620Ile | GGAACATGCTGGACAATGTGGA | TCATTGAGCAGACAGAGAAATGGAC | 149 |
| ERBB3 | 2 | p.Pro30Leu | GTAACTGGAAGAGGGCAACCAA | AGCTTGTACAGTGTCTGGTATTGG | 174 |
| ERBB3 | 28 | p.Arg1173Trp | CCCTACCCTCATGAAGTTCTTCAC | ACTCCTCATCTTCATCTTCTTCTTCAGTA | 139 |
| EXT2 | 2 | p.Arg161Trp | GATGTCTATCGCTGTGGCTTCA | CCCGGTTGATGTCATCAGTGTA | 175 |
| EXT2 | 7 | p.Val396Met | GTGAAATGAAACAAGACTGTGTGTAGAAA | CTGTCTCTGCATTTCTTCAATCTGTC | 175 |
| EXT2 | 8 | p.Arg426Gln | GCCTAACCTGGAGTTGACTATGATAGA | GGATAGATCCGGTCATTGATAATCTGC | 174 |
| FAT1 | 10 | p.Met2845Ile | AAGTTGTAATCCAGCCTGTTTCCA | ATGAGGCATTCATTGTTGAAAACCTG | 174 |
| FAT1 | 2 | p.Ile895Thr | GGTGGAATAAATGTAGGTGGGTTGT | CGTGACGGGTGTTGTTAACATC | 174 |
| FCGBP | 32 | p.Arg4950Gln | TGGGCATCGCCATCAAAGAG | TGGTTCTGTGAGTTCCTGACCTA | 169 |
| FCGBP | 16 | p.Glu2640Lys | CTGGAGAGGAGCCCACAGAACTC | CAGGCAACGCCAATGAGTTC | 172 |
| FCGBP | 8 | p.Ala1343Val | CCGAAGTCGGTCTCAATCACAA | CCCTACTCAAAGTCTACTTTCTGAAGC | 150 |
| FCGBP | 8 | p.Gly1333Arg | CCGAAGTCGGTCTCAATCACAA | CCCTACTCAAAGTCTACTTTCTGAAGC | 150 |
| FCGBP | 4 | p.Gly645Glu | CGGTCGCCCTCATAGTGTTG | GTGACCCAGCAGACGACTTCCT | 169 |
| GLI3 | 14 | p.Gly727Arg | CGATGGCACTGAGGTCTCCTAT | GTGACATTTCTTTCCTTTCCACTTGAC | 165 |
| GLI3 | 3 | p.Ser45Arg | CTGGACATTCTGTGGCTGCATA | CGAGAATGAGACCTAATTGATCATGGTTTT | 158 |
| GPLD1 | 14 | p.Asn422Ser | GGAAGCCTTCAAGGATCCTGT | CAGCTGACCTCAACCAGGAT | 174 |
| GPLD1 | 13 | p.Asp337Tyr | GACGTGCTTTTGTGACAACTGA | CACAAGTCCAATACTTTGGACAGGAA | 174 |
| ITIH5 | 13 | p.Glu757Lys | CCCACCATCCAAGATGACTCTG | ACAGTGAACGGAGAGTTAATTGGG | 150 |
| ITIH5 | 9 | p.Asn421His | GCCAATGGTGAAGATGCAGACT | ATCAGGCTCCTCAACAAGTACG | 171 |
| KDM6B | Intron6-7 | c.456+6A>G | ACTGTACGAGTCAGAGCACGATA | CTGAGCCAGAAGGCACAATCGT | 168 |
| KDM6B | Intron6-7 | c.457-8A>C | GGGACGGGATTGTACGATTGTG | TGTAGCAAGTTCCACACTTGCT | 167 |
| KDM6B | 8 | p.Pro203Ala | ACAAACGGAACTATGGAGCCAA | CTATACCCACACCTGTTCAGAGTTG | 173 |
| KDM6B | 8 | p.Glu221Asp | ACAAACGGAACTATGGAGCCAA | CTATACCCACACCTGTTCAGAGTTG | 173 |
| KDM6B | 11 | p.Ser444Gly | GGATGGGTGGAGCTTGTCTTG | CCCAAGAACGGTTTCCGAC | 161 |
| KDM6B | 11 | p.Pro482Ser | CAGTCGGAAACCGTTCTTGG | TTCTAAGATCTCTCCATCCTCTCGG | 175 |
| KDM6B | 11 | p.Pro511His | CCCGAGAGGATGGAGAGATCTT | AATCCTGAGTGCCCACAGAAAA | 138 |
| KMT2C | 8 | p.Cys391* | ACCTCTATTATATTACACAAACCTTTAGCACC | GGATATAGCGGTTACTCCATTAAAACGT | 162 |
| KMT2C | 8 | p.Arg380Leu | GCACACTTTGCACTCAGGACAT | GTTAATTATAGCGAAGGAAGATGCAAACTG | 172 |
| KMT2C | 8 | p.Asp348Asn | GCACACTTTGCACTCAGGACAT | GTTAATTATAGCGAAGGAAGATGCAAACTG | 172 |
| KMT2C | 7 | p.Pro309Ser | CCGTAGTAAACTTACATCTTTCAGGAGC | GTGCATTTTGTAAGCACCTTGGA | 174 |
| LATS2 | 8 | p.Asp1006Gly | ACCTTCGCTGGCATCGTT | CCCTTCTTCAGCGCCATTGA | 141 |
| LATS2 | 8 | p.Ala955Val | CTTCCGGATGTCACTGGAGA | AGAACACGCTCCACATTCCA | 167 |
| MACF1 | 1 | p.Ile23Met | GATTAGGAGAGGAAGGAAATGGGAAATT | CTGAGGTTTCTTCCTGGTGAGATG | 175 |
| MACF1 | 1 | p.Cys242Tyr | GTGTGGAGGAAGGAACCAAGAGT | AGAAGTAGGCAGTCTGGAATCCT | 167 |
| MACF1 | 30 | p.Arg2850Ser | GCAAGAAGGGATTGAAGTGTGTG | CCCATCAGAAACTCTCACTTTAACCT | 175 |
| MACF1 | 30 | p.Glu2978Lys | TCTCATATGAAGCAGTCTACCTCATGT | TCATTGGTTGCTGCAAAACCTG | 142 |
| MAGEC1 | 4 | p.Val176Leu | CCAGTCTGTTCTCCAGATTCCTG | CTCAATAAAGTGGAGGAGAAGGAGC | 159 |
| MAGEC1 | 4 | p.Ile178Leu | CCAGTCTGTTCTCCAGATTCCTG | CTCAATAAAGTGGAGGAGAAGGAGC | 159 |
| MAGEC1 | 4 | p.Val447Gly | TTTATTGAGTATTTTACAGAGTTCTCCTGAGAG | GGAACTCTGGAAAAGACTCAATAAAGTGT | 130 |
| MCM9 | 13 | p.Met1096Val | GTAAAGTGAAGAGGGATTCTTTGGAAAC | CCATCGGAATCCAAATCAAAATCCC | 175 |
| MCM9 | 13 | p.Ser898Phe | GATCAGTTTTGACTTCTGCTTGAAAGT | CCATCCTCAGTCCACTCCTGTA | 175 |
| MCM9 | 13 | p.Glu816Asp | GGGACATGATGAGTCAGTACTGAG | CCAAAGGAGCAAAGTGGACATTG | 165 |
| MXRA5 | 7 | p.Val2688Ile | TCCGTCTCCATCCTGCATACA | CAGGGACGTTTCTCCTGGAC | 152 |
| MXRA5 | 7 | p.Glu2426Asp | GGGTTACCGTTGATCTTGGG | TATTCAGAAAGCCCAGCGTTCTG | 126 |
| MXRA5 | 77 | p.Ile1819Leu | CTTTGAGCTGCTCTGGTGGAA | ACCTCCGTTGTTGCACACT | 148 |
| MXRA5 | 5 | p.Gly943Asp | CTCAGCCAAGGAGACAGCATCCA | CCTTATGAACCATCTCCTACTCTGCA | 165 |
| NACA | 3 | p.Asp1401Ala | GGGAGTGAGATCTCCTTTGGA | AAGGAGGTCCCGCTATGACT | 170 |
| NACA | 3 | p.Ser1377Pro | GATGGGATAGCTGGTCCTCTTTTG | CTCTAAAGGAGGCCCAACTACTC | 126 |
| NACA | 3 | p.Ser1304Pro | GGGTACCTGGGCTTCCTTTTG | CTCCCTCCCTAAAAGGAGGTCT | 168 |
| NACA | 3 | p.His1283Pro | GGGTACCTGGGCTTCCTTTTG | CTCCCTCCCTAAAAGGAGGTCT | 168 |
| NACA | 3 | p.Ser435Phe | ATGTTACTTATGGGACCTGATGACATTG | CTCTCCTAATGCCACTTATCATTATCCT | 174 |
| NACA | 3 | p.Ser435Phe | TTCCAACAGAAGAAACGGGCATT | CCCTTCTGGCTCCTTAAATGTAGC | 131 |
| NACA | 3 | p.Val381Asp | TGAGAATGAGAGAGGTTGTAGGAGATAATG | AGAAATGAGGTAGTTCCTGCTACTGT | 160 |
| NCOA2 | 20 | p.Gly1363Arg | TGTCACAGTGGCGGTATGAAAA | GTCCCATGATGCAACAGTCTCA | 133 |
| NCOA2 | 11 | p.Glu653Val | GTATCCGACAAAGAGCTGGCTAA | CTGGAGAGCAAAAGGAAACAAATGACC | 175 |
| NCOA2 | 11 | p.Ala407Ser | CCATTTGTTCCTTTGGGCCATT | AATGTGTGTGTGATGAATCCGGAT | 172 |
| NUMA1 | 22 | p.Tyr1836His | CTGAGTTGCCATAATCGGGAGAA | GCAACAGATTCACTCTGTCTTCCA | 167 |
| NUMA1 | 21 | p.Lys1766Arg | CAGGGATGGGAGTGAAGTAGAGA | AGGTTTCCTTTCCATAGCAAGCT | 139 |
| NUMA1 | 15 | p.Gln1089Glu | CCAGATGCGTGCTCCTTTTCT | ATGCCCTGACGGAAAAGGAAG | 151 |
| PER1 | 22 | p.Ala1196Val | CCCTTGAACTTGAGCTCAATTCT | CCTCGGTTTTCTGAGGACCA | 206 |
| PER1 | 19 | p.Thr866Pro | GAGAGAACACTGGGAGAGGGTA | CTGCCGATCCAAAGCCAAGC | 170 |
| PER1 | 6 | p.Val240Ile | GATGGAGCAGTGGAACCATAGAA | CCTGCCAGGATACCTTCTCAGT | 175 |
| RABL6 | 9 | p.Pro373Thr | GGCGCAGCATCATCTCTAGG | GCGCTCCGAAATCCAGGAC | 145 |
| RABL6 | 10 | p.Thr387Met | GTTCTGGTGCCGAGTGAAGA | GGTTGTGTCTTCCAGGAAGCTG | 141 |
| RABL6 | 15 | p.Arg375fs | CCGATGAGCTGGAGGCTTT | GCGATGGTACAGAGGCAAATG | 160 |
| ROS1 | 21 | p.Ser1054Arg | CCCATTTTCATGCTTAGGTTTGTTCC | CTCTACCTGTGCCTCTACTTTTCA | 138 |
| ROS1 | 18 | p.Arg894Trp | ACTGATTAAATCTGGCTGGTTCCAA | GGATACCACCATCACAGAATTTGC | 173 |
| ROS1 | 16 | p.Trp827Leu | CTGCTTTAAGTACTCACAATAAGCGAGA | CTCTATTCAGTGGAAAGCACCAGA | 175 |
| RRBP1 | 5 | p.Asn727Ile | CTCTGAGGACGGGAGAGGACAA | CTGGAAGAGAAGGAAAAACTGCTG | 131 |
| RRBP1 | 3 | p.Gln606His | CTCTGGGCCACATCTGTATTTCT | CACAAAGGTAGAGGGTATTACAAACCAG | 174 |
| RRBP1 | 3 | p.Gln606His | ATTTAAAGACAGCCTTTCCTTCTAAGTTCA | GGGTAAAAAGACAGAGTCAGCTTCT | 175 |
| RRBP1 | 3 | p.Lys210Arg | CTCTGCCTTTTTCCCTTGGTTTG | TGGCACTACTCAGGGCAAAAAG | 148 |
| RRBP1 | 3 | p.Asp85Glu | GGTTCTCGAAGGAGGACAGTCA | GTCGAGAAGAAAAAGAAGGAGAAAACAGTG | 128 |
| SCUBE2 | 21 | p.His107Arg | ATGTGTGCTTGTCAGCAGTTTG | GTCAAAGAAGCTGTGGATTCAGTTC | 175 |
| SCUBE2 | 20 | p.Asn835Asn | CTGGGTAATTGCCTGGGTAGTT | GGTGGTCAGGACAGAGATGACA | 138 |
| SCUBE2 | 19 | p.Asn790Thr | TTTTTCCAAATTCAGGCTGGTATGTT | CAAACTTTAAAATCTTTGTGCTGCATTTGA | 172 |
| SDHA | 11 | p.Thr508Ile | TTTTTGTTTTAGGAGATAAAGTCCCTCCAA | CGAGTCCAGGCTCTTACCTTCT | 148 |
| SDHA | 15 | p.Leu649fs | GCTTAACTTACCACTGACTCTTCTTTTCAA | CACATCTTGTCTCATCAGTAGGAGC | 132 |
| SDHA | 15 | p.Cys654Arg | GCTTAACTTACCACTGACTCTTCTTTTCAA | CACATCTTGTCTCATCAGTAGGAGC | 132 |
| SEC16A | 1 | p.Arg641Cys | GCTTCCGTGGTGGAGTTAAGAG | GCCAACGTGGTTGGTGAAGTAA | 170 |
| SEC16A | 1 | p.Arg346His | CCCTTGGAAAAACATCGCCAG | AGGAGTGAAGAATGAGCACCG | 172 |
| SH2B3 | 1 | p.Leu6Pro | GGTGGAGCTCAGAAGGACAT | GATTCAGAAGAAGAGGTGAGAGCAA | 130 |
| SH2B3 | 2 | p.Trp262Arg | AAGAGCATCAGGAACAAGCCTT | GGGTGTGAAAAGCCTTGTCACT | 173 |
| SH2B3 | 7 | p.Asn537Asp | TCTCCCAGGGCGATCCTCAC | GGATGAGTCCATTTCGTAGTCCG | 139 |
| SLC25A5 | 2 | p.Phe82Ile | CATTATAGACTGCGTGGTCCGTA | CCAAAACTGGGTTCTCTTGTCCA | 172 |
| SLC25A5 | 3 | p.Arg244Cys | GATGATCGCACAGACTGTCACT | CCCACGACTACAACTTTATCTTCTGC | 139 |
| TACC2 | 4 | p.Arg960Lys | AAGAGTCAGAATTGTCAGCACCAA | CCAGTGCAGCAAGTTTCCTTTC | 175 |
| TACC2 | 4 | p.Pro1563Arg | CTGGAGAGGAGCAGGCAGGAATT | GCTTTCCAGAAGGAATTCTGTCTTG | 151 |
| TNN | 6 | p.Arg440Ser | GGGACTGAGTATAAGATCACGGT | GAGAGGAGTCAGCCAAGAAAAATATGA | 154 |
| TNN | 15 | p.Val1073Ile | CCTTAAAATAGGAAGAAACTCCACCTCTT | GCCATGCAGGTAGATGGTGTAC | 168 |
| TNN | 16 | p.Val1130Met | TCATCTTCCTTCTCAGGTCTTCCA | GCTTGTCCTGGGAGAAATCACC | 172 |
| TNN | 16 | p.Asp1135Glu | TCATCTTCCTTCTCAGGTCTTCCA | GCTTGTCCTGGGAGAAATCACC | 172 |
| TNN | 17 | p.Arg1157Gln | TACTCGCACACATGGGTTGATT | CTGGAGGCCACTTGGAAGAAAT | 173 |

**Suppl Table 3B Primer sequence information for SV validation**

| **Fusion gene** | **5'-3' forward sequence** | **5'-3' reverse sequence** | **Product size (bp)** |
| --- | --- | --- | --- |
| FSIP1-BAZ2A | GCTATATGGCAGACTAGATGT | ACTTGTTGGTGTTGATGGT | 214 |
| SET-DPP10 | CCTTGAGCAGAATACAATGG | CAAGACTATCCTGGCTAACA | 233 |
| MUC19-ORC5 | CTCAGAGAAGACAGGCATAT | GGTTCACGCCATTATCCT | 325 |

**Suppl Table 4 Antibody information**

| **Gene name** | **Vendor** | **Item number** | **titration** |
| --- | --- | --- | --- |

| CDK4 | Cell Signaling | 127905 | 1;50 |
| --- | --- | --- | --- |
| BRAP (D-5) | Santa Cruz Biotechnology,INC | sc-166012 | 1;100 |
| FOP (B-1) | Santa Cruz Biotechnology,INC | sc-374340 | 1;100 |
| TERT(A-6) | Santa Cruz Biotechnology,INC | sc-393013 | 1;100 |
| Limd1 (H-4) | Santa Cruz Biotechnology,INC | sc-271448 | 1;100 |
| Msi1(69-Q) | Santa Cruz Biotechnology,INC | sc-135721 | 1;100 |
| Y14(4C4) | Santa Cruz Biotechnology,INC | sc-32312 | 1;100 |
| TBRG1(D-9) | Santa Cruz Biotechnology,INC | sc-515620 | 1;100 |
| Mi2(B-4) | Santa Cruz Biotechnology,INC | sc-55606 | 1;100 |
| GADD153 (H-5) | Santa Cruz Biotechnology,INC | sc-166682 | 1;100 |
| Myosin X (C-1) | Santa Cruz Biotechnology,INC | sc-166720 | 1;100 |
| Epac (A-5) | Santa Cruz Biotechnology,INC | sc-28366 | 1;500 |
| ErbB-3 (RTJ.2) | Santa Cruz Biotechnology,INC | sc-415 | 1;200 |
| HDGF (E-7) | Santa Cruz Biotechnology,INC | sc-271344 | 1;1000 |
| KIF14 (E-3) | Santa Cruz Biotechnology,INC | sc-365553 | 1;500 |
| NDUFB6 (B-2) | Santa Cruz Biotechnology,INC | sc-515596 | 1;500 |
| cathepsin K (E-7) | Santa Cruz Biotechnology,INC | sc-48353 | 1;200 |
| PP2A-Aa/b (4G7) | Santa Cruz Biotechnology,INC | sc-13600 | 1;200 |
| 14-3-3 e (8C3) | Santa Cruz Biotechnology,INC | sc-23957 | 1;500 |
| BAP1 (C-4) | Santa Cruz Biotechnology,INC | sc-28383 | 1;200 |
| Atm (G-12) | Santa Cruz Biotechnology,INC | sc-377293 | 1;500 |
| B-catenin (12F7) | Santa Cruz Biotechnology,INC | sc-59737 | 1;200 |
| NF-1 (D-2) | Santa Cruz Biotechnology,INC | sc-74444 | 1;200 |
| NuMA (F-11) | Santa Cruz Biotechnology,INC | sc-365532 | 1;100 |
| Positive cofactor 4 (H-12) | Santa Cruz Biotechnology,INC | sc-166280 | 1;2000 |
| MDM2 IF2 | Invitrogen | 182403 | 1;200 |
| HDAC5 (HDAC5-35) | abcam | ab50001 | 1;1000 |
| Cyclin D1 | Epitomics | AC-0017 | 1;100 |
| ER (SP1) | Thermo scientific | RM-9101 | 1;200 |
| P16 | BIOCARE | ACR3007C | 1;50 |
| Ki67 | Dako/Agilent | M7240 | 1;100 |
| Bcl-2 | Dako/Agilent | M0887 | 1;50 |

**Suppl Table 5 Chromosomal CNV and associated genes**

| Chromosome and region | | | Start | End | Case number | Frequency(%) | Specific genes |
| --- | --- | --- | --- | --- | --- | --- | --- |
| Frequently Gained | | | | | | | |
| 1 | | 1q21.1 | 144000656 | 147380560 | AS3,AS6, | 20 | RNF115,LIX1L, PIAS3, **RBM8A**, CHD1L,BCL9, NBPF10 |
|  |  | 1q21.2-q21.3 | 149720001 | 151878880 | AS2,AS3,AS4,AS5,AS6,AS7 | 60 | ANP32E,BNIPL,**CTSK**,CTSS,ECM1,GOLPH3L, HORMAD1,MCL1,PLEKHO1, PRUNE, PSMB4, SELENBP1,SETDB1,SF3B4,THEM4,TUFT1 |
|  |  | 1q21.3-q23.1 | 153360001 | 157190000 | AS2,AS3,AS4,AS5,AS6,AS7 | 60 | ADAR,CCT3,**HDGF**,ILF2,IQGAP3,MEF2D,NPR1, NTRK1,PBXIP1,PRCC, PYGO2,RAB25,SCAMP3,SEMA4A,SLC39A1,UBE2Q1,YY1AP1 |
|  |  | 1q32.1,q32.2 | 199850001 | 207300000 | AS2,AS3,AS5,AS6 | 40 | CYB5R1,IKBKE,**KIF14**,KLHDC8A,LGR6,MAPKAPK2,MDM4,NFASC,NUAK2, NUCKS1,PIK3C2B,PKP1,PPFIA4,PTPN7,TIMM17A,UBE2T |
|  | | 1q42.2,q42.3 | 234450001 | 234910000 | AS1,AS3,AS6 | 30 | TARBP1 |
|  | | 1q44 | 244340001 | 247650000 | AS3,AS5,AS6 | 30 | DESI2,KIF26B,SMYD3 |
| 5 | | 5p15.33 | 10001 | 1960000 | AS1,AS2,AS7 | 30 | **TERT**,NKD2,CEP72,CLPTM1L,LPCAT1,SDHA,TPPP |
|  |  | 5p15.1 | 16490001 | 17670000 | AS2,AS4,AS5,AS7 | 40 | **MYO10**, BASP1, RETREG1 |
|  |  | 5p13.3 | 31300001 | 32650000 | AS2,AS4,AS5,AS7 | 40 | GOLPH3, PDZD2, **SUB1**, ZFR, CDH6 |
|  | | 5q13.1,q13.2 | 68260001 | 70900000 | AS2,AS7 | 20 | CCNB1,CDK7, CENPH, MARVELD2, RAD17,TAF9 |
| 6 | | 6q24.3-q25.2 | 148600001 | 154580000 | AS2,AS5 | 20 | SASH1, AKAP12, C6orf211, CCDC170, ESR1, PCMT1, ZBTB2, MYCT1, RGS17 |
|  | | 6q25.2-25.3 | 154580001 | 155590000 | AS2,AS5 | 20 | TIAM2 |
|  | | 6q25.3 | 157110001 | 159360000 | AS2,AS5 | 20 | ARID1B, EZR, GTF2H5, SNX9, SYNJ2, ZDHHC14 |
| 7 | | 7p22.3-p22.1 | 50001 | 7050000 | AS2,AS4,AS5,AS7 | 40 | MAFK, CYP2W1, GPER1, MICALL2, PDGFA |
|  | | 7p12.3 | 47200001 | 48010000 | AS2,AS4,AS7 | 30 | TNS3, HUS1 |
|  | | 7p12.2-12.1 | 50530001 | 51750000 | AS2,AS7 | 20 | GRB10 |
| 8 | | 8P11.22 | 37360001 | 39070000 | AS2,AS3,AS5,AS7 | 40 | ADAM9, BAG4, EIF4EBP1, **FGFR1**, GPR124, HTRA4, NSD3, PPAPDC1B, PROSC, RAB11FIP1, TACC1 |
|  | | 8q13.1 | 67320917 | 68343504 | AS2,AS3,AS4,AS5,AS7 | 50 | ARMC1,MYBL1,SGK3,CSPP1,COPS5 |
|  | | 8q13.3 | 70750001 | 71020000 | AS2,AS3,AS4,AS5,AS7 | 50 | PRDM14,NCOA2 |
|  | | 8q21.11 | 73902397 | 74954388 | AS2,AS3,AS4,AS5,AS7 | 50 | TERF1 |
|  | | 8q24.21 | 128671900 | 129171700 | AS2,AS3,AS4,AS5,AS7 | 50 | PVT1,MYC |
|  | | 8q24.21 | 131035000 | 131224400 | AS2,AS4,AS7 | 30 | ASAP1 |
| 12 | | 12p13.33-p13.31 | 150001 | 9130000 | AS2,AS3,AS5,AS7 | 40 | **CCND2**, **CHD4**, ERC1, KDM5A, TEAD4, RAD52, ACRBP, CDCA3, PTPN6, RAD51AP1, NANOG, WNT5B |
|  |  | 12q13.11-q13.13 | 48110001 | 54900000 | AS2,AS3,AS5,AS7 | 40 | ARF3, BCDIN3D, CCNT1, CERS5, FMNL3, GALNT6, HDAC7, ITGA5, MCRS1, NR4A1, PCBP2, **RAPGEF3**, RARG, SMAGP, TMBIM6 |
|  |  | 12q13.2-q14.1 | 56030001 | 58310000 | AS2,AS3,AS4,AS5,AS7 | 50 | CDK2, **CDK4**, **DDIT3**, DGKA, **ERBB3**, GLI1, ITGA7, MMP19, NABP2, PA2G4, RAB5B, SHMT2, STAT2, STAT6, TIMELESS, TSPAN31(SAS) |
|  |  | 12q14.1-q14.3 | 62810001 | 67700000 | AS2,AS4,AS5,AS7 | 40 | **HMGA2** |
|  |  | 12q15.1 | 68611133 | 70187177 | AS2,AS3,AS4,AS5,AS7 | 50 | **MDM2**, CPM, NUP107, RAP1B, SLC35E3, LYZ, FRS2, CCT2, YEATS4 |
|  |  | 12q23.3-q24.21 | 108970001 | 114480000 | AS2,AS3,AS5,AS7 | 40 | ALDH2, ANAPC7, **BRAP**, CORO1C, MAPKAPK5, PPP1CC, PTPN11, RPL6, SH2B3, TCTN1, TMEM119, TRPV4 |
|  |  | 12q24.21-q24.23 | 116670001 | 118970000 | AS2,AS3,AS5,AS7 | 40 | PEBP1, WSB2 |
|  |  | 12q24.23-q24.31 | 120080001 | 125670000 | AS2,AS3,AS4,AS5,AS7 | 50 | DENR, KDM2B, **MSI1**, PXN, RAB35, RNF34, SETD1B, SETD8, TRIAP1 |
|  |  | 12q24.33 | 128790001 | 133500000 | AS2,AS3,AS4,AS5,AS7 | 50 | DDX51, MMP17, **RAN**, ULK1 |
| 17 | | 17p13.3,p13.1 | 1 | 10300000 | AS2,AS4,AS5,AS7 | 40 | ARHGEF15, AURKB, C1QBP, DHX33, FAM57A, FBXO39, MINK1, NLRP1, NUP88, PELP1, RCVRN, RPAIN, SERPINF1, TAX1BP3, TRPV3, WDR16, WRAP53, **YWHAE** |
|  |  | 17p12,p11.2 | 15580001 | 21570000 | AS2,AS4,AS5,AS7 | 40 | ADORA2B, COPS3, EPN2, SHMT1, SPECC1, SREBF1, **TRPV2**, ULK2, USP22 |
|  |  | 17q12,q21.1 | 36290001 | 39850001 | AS2,AS4,AS7 | 30 | CDC6, CDK1, **ERBB2**, FBXL20, IGFBP4, KRT23, MIEN1, PIP4K2B, PLXDC1, RPL23, SMARCE1, TBC1D3, TNS4, TOP2A |
|  |  | 17q21.2 | 39850001 | 40530000 | AS2,AS4,AS5,AS7 | 40 | ACLY, **FKBP10**, LEPREL4, RAB5C, STAT3, STAT5A, STAT5B |
|  |  | 17q21.2,q21.31 | 40530001 | 43390000 | AS2,AS4,AS7 | 30 | ETV4, **HDAC5**, HSD17B1, KIF18B, NBR1, NMT1, RUNDC1, VAT1 |
|  |  | 17q21.32 | 45530001 | 46220000 | AS2,AS5 | 20 | CBX1, KPNB1, OSBPL7, SP2, CDK5RAP3 |
|  |  | 17q21.33 | 48060001 | 48410000 | AS1,AS2 | 20 | **ITGA3**, SAMD14, PDK2 |
|  |  | 17q22,q24.2 | 55450001 | 63200000 | AS2,AS5 | 20 | MSI2, **TRIM37**, APPBP2, BCAS3, BRIP1, CLTC, DDX5, GNA13, **MED13**, PPM1D, RPS6KB1, TBX2, TLK2, USP32, MAP3K3 |
|  |  | 17q23.2,q23.3 | 60710001 | 61220000 | AS1,AS2,AS5 | 30 | **MRC2** |
|  |  | 17q24.2 | 64620001 | 66440000 | AS2,AS5 | 20 | BPTF, **KPNA2**, PRKCA |
|  |  | 17q25.1,q25.3 | 70100001 | 81110000 | AS2,AS4,AS5,AS7 | 40 | MRPL58, RAB40B, SOX9, **BIRC5**, FOXK2, JMJD6, RAC3, TK1, TTYH2,USP36 |
| Frequently Lost | | | | | | | |
| 3 | 3p22.1-P21.1 | | 41110001 | 53310000 | AS3,AS6 | 20 | ACY1,ALS2CL,APEH,CISH,**CTNNB1**,CYB561D2,DAG1,**LIMD1**,MST1,PLXNB1,RASSF1,RNF123,SETD2,SMARCC1,TCTA,TMEM115,TUSC2,UBA7,USP4 |
|  | 3p21.1 | | 52420001 | 52670000 | AS2,AS3,AS5,AS6 | 40 | **BAP1**, NISCH, SEMA3G, PBRM1 |
|  | 3q13.31 | | 115230001 | 116910000 | AS5,AS6,AS8,AS9 | 40 | GAP43, LSAMP, LSAMP-AS1, TUSC7 |
|  | 3q22.3 | | 135730001 | 136370000 | AS5,AS6 | 20 | MSL2, STAG1 |
|  | 3q25.33,q26.1 | | 160390001 | 161120000 | AS3,AS5 | 20 | PPM1L |
| 9 | 9P23-22.3 | | 9530001 | 14800000 | AS5,AS6 | 20 | PTPRD, LURAP1L, MPDZ, **NFIB**, TYRP1 |
| 9  11 | 9p22.2-p22.1 | | 17680001 | 18890000 | AS5,AS6 | 20 | SH3GL2 |
|  | 9p22.1,p21.3 | | 19450001 | 23780000 | AS5,AS6 | 20 | **CDKN2A**, CDKN2B, MTAP |
|  | 9p21.1 | | 30900001 | 32870000 | AS5,AS6 | 20 | **NDUFB6** |
|  | 11q13.3-q22.3 | | 69360001 | 104810001 | AS5,AS6, | 20 | B3GNT6,**CCND1**,ENDOD1,**NUMA1**, USP35,UVRAG |
| 11 | 11q22.3-q23.3 | | 104810001 | 116180000 | AS4,AS5,AS6 | 30 | **ATM**,BCO2,BTG4,CUL5,**PPP2R1B**, SDHD, ZBTB16 |
|  | 11q23.3-q24.3, | | 120860001 | 127950000 | AS4,AS5,AS6 | 30 | BLID,EI24,HEPACAM,**TBRG1**,VWA5A,HEPN1 |

**Suppl Table 6 Immunohistochemistry analysis of selected oncogene/tumor suppressor genes product expression in Müllerian adenosarcoma**

|  | MAS | | EM | | | (MAS vs. EM) | (cervix vs. uterus vs. ovary) | |  |
| --- | --- | --- | --- | --- | --- | --- | --- | --- | --- |
| No Cases | 29 | | 8 | | |  |  |  |  |
| Markers | Median | Range (95% CI) | | Median | Range (95% CI) | P value | P value | | |
| ATM | 100 | 66-132 | | 80 | 38--137 | 0.89 | 0.86 | | |
| BAP1 | 100 | 93--168 | | 0 | 1--133 | 0.15 | 0.88 | | |
| BCL-2 | 100 | 46--121 | | 0 | 0-4 | **0.006** | 0.30 | | |
| CCND1 | 50 | 46--121 | | 0 | 3 | **0.002** | 0.21 | | |
| CDK4 | 30 | 42--107 | | 5 | 0--20 | **0.001** | 0.73 | | |
| EPAC | 140 | 90--186 | | 210 | 153--252 | 0.56 | 0.24 | | |
| ER | 100 | 68--123 | | 100 | 62--138 | **0.03** | 0.28 | | |
| ERBB3 | 100 | 95--182 | | 200 | 112--221 | 0.72 | 0.55 | | |
| FOPB1 | 200 | 182--250 | | 200 | 172--237 | 0.16 | 0.42 | | |
| HDGF | 50 | 13--79 | | 10 | 0--37 | 0.79 | 0.26 | | |
| HMGA2 | 10 | 9--21 | | 5 | 2--13 | **0.005** | 0.07 | | |
| Ki-67 | 100 | 74--132 | | 50 | 14--49 | **0.04** | 0.20 | | |
| KIF14 | 30 | 21--73 | | 0 | 17 | **<0.001** | 0.60 | | |
| MDM2 | 100 | 104--153 | | 160 | 60--168 | **0.02** | **0.05** | | |
| MSI1 | 50 | 27--104 | | 50 | 0-112 | 0.62 | 0.46 | | |
| MYOSIN X | 100 | 69--138 | | 50 | 14--88 | 0.79 | 0.26 | | |
| NDUFB6 | 150 | 123--199 | | 125 | 71--175 | 0.08 | **0.04** | | |
| NF1 | 200 | 129--199 | | 100 | 51-171 | 0.1 | 0.28 | | |
| NUMA | 300 | 231--282 | | 200 | 188--256 | 0.32 | 0.73 | | |
| PCO4 | 100 | 96--150 | | 100 | 95--172 | 0.17 | 0.69 | | |
| P16INK4a | 125 | 96--160 | | 50 | 4--96 | **0.04** | 0.17 | | |
| PP2A | 280 | 255--303 | | 267 | 228--305 | 0.37 | 0.37 | | |
| RAN | 170 | 138--200 | | 100 | 69--140 | 0.8 | 0.51 | | |
| YWHAE | 100 | 66-132 | | 80 | 38--137 | **0.006** | 0.91 | | |

|  |  |  |  |  | 150 |
| --- | --- | --- | --- | --- | --- |

MAS: Müllerian adenosarcoma; EM: endometrium.

**Suppl Table 7 Frequent structure variation in Müllerian adenosarcoma**

| Frequency | Case# | Breakend 1 Chrom | Breakend 1 Pos | Breakend 1 Gene Name | Breakend 2 Chrom | Breakend 2 Pos | Breakend 2 Gene Name | Breakend 1 Depth | Breakend 2 Depth |
| --- | --- | --- | --- | --- | --- | --- | --- | --- | --- |
| 2 | AS3,AS7 | chr1 | 17226400 | None | chr4 | 160898604 | None | 155 | 81 |
| 2 | AS5,AS7 | chr1 | 17784557 | None | chr2 | 210523447 | MAP2 | 105 | 99 |
| 2 | AS5.AS8 | chr1 | 88336599 | None | chr16 | 83666361 | CDH13 | 103 | 48 |
| 6 | AS3-5,AS8 | chr1 | 109494853 | CLCC1 | chr3 | 110413394 | None | 111 | 51 |
| 4 | AS1,AS2,AS4,AS9 | chr1 | 110191247 | None | chr3 | 155531847 | None | 111 | 47 |
| 6 | AS3-5,AS7,AS8,AS10 | chr1 | 121215018 | None | chr5 | 49771063 | None | 40 | 115 |
| 4 | AS2,AS5,AS6,AS8 | chr1 | 147855727 | None | chrX | 92418807 | None | 158 | 119 |
| 4 | AS5 | chr1 | 148928247 | None | chr2 | 91848017 | None | 200 | 84 |
| 6 | AS1,AS3,AS5,AS6-8 | chr1 | 149216152 | None | chr15 | 75867322 | PTPN9 | 205 | 146 |
| 2 | AS3,AS7 | chr1 | 163314432 | NUF2 | chr5 | 55384182 | None | 117 | 111 |
| 5 | AS3,AS5,AS6,AS8,AS10 | chr1 | 168024586 | DCAF6 | chr19 | 24033179 | ZNF254 | 111 | 55 |
| 3 | AS2,AS3,AS9 | chr1 | 168186489 | DCAF6 | chr3 | 53175885 | ZNF83 | 104 | 78 |
| 4 | AS1,AS4,AS5,AS7 | chr1 | 180903258 | KIAA1614 | chr3 | 48537167 | SHISA5 | 92 | 64 |
| 2 | AS6,AS10 | chr1 | 199440227 | None | chr5 | 33633345 | ADAMTS12 | 120 | 65 |
| 8 | AS2-8,AS10 | chr1 | 207579635 | None | chr6 | 57494241 | LOC100996486 | 95 | 169 |
| 5 | AS3,AS5,AS6,AS9,AS10 | chr1 | 214656661 | PTPN14 | chr5 | 115177797 | AP3S1 | 121 | 41 |
| 3 | AS3,AS6,AS7 | chr1 | 223293291 | TLR5 | chr5 | 54401983 | GZMA | 73 | 129 |
| 3 | AS3,AS4,AS7 | chr1 | 246565670 | SMYD3 | chr21 | 45327478 | AGPAT3 | 99 | 67 |
| 6 | AS2,AS4-8 | chr10 | 38908595 | None | chr22 | 16884260 | None | 28 | 176 |
| 4 | AS2,AS3,AS5,AS7 | chr10 | 42646416 | None | chr16 | 32097315 | None | 72 | 151 |
| 4 | AS1,AS3,AS4,AS5 | chr10 | 55983281 | PCDH15 | chr13 | 66704573 | None | 94 | 118 |
| 3 | AS4,AS5,AS7 | chr10 | 117006340 | ATRNL1 | chrX | 128670972 | None | 119 | 117 |
| 6 | AS1-3,AS5,AS8,AS10 | chr11 | 61841813 | None | chr14 | 81786774 | STON2 | 111 | 80 |
| 2 | AS7,AS8 | chr11 | 108585748 | DDX10 | chr13 | 21750661 | SKA3 | 82 | 129 |
| 2 | AS3,AS8 | chr12 | 14476864 | None | chrX | 38058552 | SRPX | 45 | 105 |
| 2 | AS5,AS8 | chr13 | 21490883 | None | chr17 | 7711989 | DNAH2 | 71 | 129 |
| 5 | AS1,AS3,AS4,AS7,AS8,AS10 | chr13 | 35686543 | NBEA | chr15 | 20873307 | None | 71 | 179 |
| 8 | AS1-4,AS6,AS8-10 | chr13 | 63621212 | None | chr17 | 21666609 | None | 156 | 67 |
| 10 | AS1-10 | chr13 | 63649022 | None | chr20 | 26149746 | None | 132 | 135 |
| 4 | AS3,AS6,AS7,AS10 | chr13 | 90550561 | None | chr15 | 52589547 | None | 103 | 115 |
| 3 | AS1,AS3,S4,AS9 | chr14 | 20301314 | None | chr15 | 22419158 | None | 123 | 56 |
| 2 | AS3,AS4 | chr14 | 106484215 | IGH | chr15 | 22486776 | LOC642131 | 106 | 71 |
| 4 | AS1.AS6,AS9,AS10 | chr15 | 20000101 | None | chr21 | 14345806 | None | 138 | 118 |
| 5 | AS3,AS4,AS7,AS9,AS10 | chr15 | 20014813 | None | chr21 | 14362821 | None | 187 | 162 |
| 8 | AS3,AS8,AS10 | chr15 | 20015855 | None | chr21 | 14363011 | None | 113 | 136 |
| 3 | AS3,AS4,AS7 | chr15 | 20452695 | RHPN2P1 | chr16 | 33406290 | None | 183 | 139 |
| 4 | AS4,AS5,AS8,AS9 | chr15 | 20470698 | RHPN2P1 | chr19 | 33506264 | None | 130 | 134 |
| 5 | AS3-5,AS8,AS9 | chr17 | 21525277 | None | chr20 | 26106896 | None | 188 | 112 |
| 9 | AS1,AS2, AS3-10 | chr19 | 19632427 | NDUFA13 | chr22 | 16347241 | NF1P6 | 122 | 93 |
| 7 | AS2,AS3,AS5-10 | chr2 | 13534907 | None | chr15 | 20609640 | None | 147 | 119 |
| 7 | AS2,AS3,AS5,AS6,AS8,AS10 | chr2 | 13534941 | None | chr15 | 20609594 | None | 111 | 63 |
| 7 | AS1,AS2,AS4-6,AS8,AS9 | chr2 | 13535013 | None | chr15 | 20609591 | None | 111 | 65 |
| 2 | AS3,AS7 | chr2 | 54111546 | PSME4 | chr14 | 40317376 | None | 112 | 79 |
| 5 | AS1-6 | chr2 | 71668034 | None | chr14 | 98494391 | None | 106 | 88 |
| 3 | AS2,AS5,AS10 | chr2 | 78091920 | None | chr4 | 174044215 | None | 123 | 100 |
| 2 | AS3,AS8 | chr2 | 91634988 | LOC101060169 | chr10 | 42645482 | None | 151 | 150 |
| 2 | AS9,AS10 | chr2 | 91694630 | None | chr22 | 17041903 | None | 157 | 85 |
| 4 | AS4-6,AS8 | chr2 | 117853382 | None | chr10 | 3138988 | PFKP | 100 | 54 |
| 2 | AS7,AS8 | chr2 | 132800294 | None | chr17 | 25285558 | None | 124 | 191 |
| 2 | AS4,AS5 | chr2 | 133011818 | None | chr16 | 33964361 | None | 166 | 177 |
| 4 | AS1,AS2,AS7,AS8 | chr2 | 242634320 | None | chr5 | 139347431 | NRG2 | 75 | 109 |
| 2 | AS2,AS3 | chr2 | 243052365 | LOC728323 | chr8 | 376926 | FBXO25 | 153 | 53 |
| 10 | AS1-10 | chr20 | 11281556 | None | chrX | 81651368 | None | 56 | 116 |
| 2 | AS7,AS10 | chr20 | 26252024 | None | chr22 | 17239016 | None | 105 | 105 |
| 3 | AS5-8 | chr3 | 49218384 | C3orf84 | chrX | 32001083 | DMD | 79 | 100 |
| 7 | AS3,AS4,AS7-10 | chr3 | 75994837 | ROBO2 | chr20 | 26203323 | None | 147 | 197 |
| 7 | AS1-5,AS7,AS9 | chr3 | 144687884 | None | chr22 | 18047371 | SLC25A18 | 114 | 60 |
| 3 | AS4-6 | chr3 | 151086531 | MED12L | chr12 | 118571033 | None | 80 | 61 |
| 8 | AS1-6,AS8,AS9 | chr3 | 151148544 | MED12L | chr5 | 39787751 | None | 59 | 133 |
| 2 | AS2,AS6 | chr4 | 33837359 | None | chr7 | 57608379 | None | 145 | 121 |
| 4 | AS5,AS6,AS8,AS10 | chr4 | 33837543 | None | chr20 | 26145998 | None | 115 | 109 |
| 3 | AS8-10 | chr4 | 33839916 | None | chr7 | 57610959 | None | 195 | 164 |
| 2 | AS1,AS4 | chr4 | 33857884 | None | chr20 | 26129184 | None | 97 | 113 |
| 5 | AS3,AS4,AS5,AS9,AS10 | chr4 | 33859622 | None | chr20 | 26128596 | None | 128 | 148 |
| 6 | AS2-5,AS7,AS10 | chr4 | 175884836 | ADAM29 | chr7 | 62473468 | None | 112 | 140 |
| 2 | AS3.AS6 | chr4 | 190897122 | None | chr9 | 141081917 | None | 130 | 148 |
| 4 | AS3,AS6-8 | chr5 | 5595243 | None | chr15 | 52589532 | None | 88 | 145 |
| 8 | AS1-3,AS5-7,AS9,AS10 | chr5 | 21573432 | GUSBP1 | chr6 | 57575919 | None | 77 | 120 |
| 2 | AS7,AS8 | chr5 | 39787751 | None | chr9 | 91774009 | SHC3 | 123 | 52 |
| 3 | AS2,AS3,AS10 | chr5 | 54402018 | GZMA | chr11 | 37466540 | None | 103 | 39 |
| 3 | AS4,AS7,AS8 | chr5 | 55931840 | None | chr6 | 57444972 | LOC100996481 | 66 | 160 |
| 8 | AS2-4,AS6-10 | chr5 | 141456960 | None | chr13 | 82367698 | None | 120 | 69 |
| 3 | AS2,AS5,AS7 | chr5 | 176753871 | None | chr7 | 157149907 | DNAJB6 | 129 | 48 |
| 7 | AS2-4,AS6,AS7,AS9,AS10 | chr6 | 382459 | None | chr16 | 33428528 | None | 117 | 101 |
| 3 | AS1,AS2,AS5 | chr6 | 147371567 | STXBP5-AS1 | chr19 | 13848429 | CCDC130 | 115 | 68 |
| 2 | AS7,AS8 | chr7 | 682398 | PRKAR1B | chr8 | 127865467 | None | 89 | 104 |
| 8 | AS2-8,AS10 | chr7 | 6616907 | None | chr9 | 132621533 | USP20 | 104 | 51 |
| 7 | AS2-8 | chr7 | 26241365 | CBX3 | chr15 | 40854180 | C15orf57 | 102 | 103 |
| 6 | AS2-6,AS8 | chr7 | 26252971 | CBX3 | chr15 | 40854194 | C15orf57 | 48 | 103 |
| 6 | AS1,AS6-10 | chr7 | 57606290 | None | chr13 | 63637902 | None | 105 | 179 |
| 7 | AS1-5,AS8,AS10 | chr7 | 57608639 | None | chr20 | 26145943 | None | 106 | 151 |
| 10 | AS1-10 | chr7 | 57638746 | None | chr17 | 21515211 | None | 148 | 91 |
| 2 | AS5,AS9 | chr7 | 57638967 | None | chr17 | 20772250 | CCDC144NL | 145 | 119 |
| 3 | AS4-6 | chr7 | 57706901 | None | chr20 | 25834871 | None | 100 | 121 |
| 7 | AS2-4,AS7 | chr7 | 57729226 | None | chr20 | 26248049 | None | 114 | 156 |
| 7 | AS6,AS7,AS9,AS10 | chr7 | 57730253 | None | chr20 | 25864477 | None | 118 | 108 |
| 4 | AS6,AS7,AS9,AS10 | chr7 | 57732769 | None | chr20 | 26244216 | None | 180 | 105 |
| 6 | AS1,AS8-10 | chr7 | 61891240 | None | chr16 | 32543549 | None | 122 | 82 |
| 6 | AS5,AS8,AS9 | chr7 | 61891341 | None | chr16 | 32543415 | None | 95 | 168 |
| 4 | AS2,AS3,AS5,AS6 | chr7 | 103814591 | ORC5 | chr12 | 40832785 | MUC19 | 142 | 98 |
| 4 | AS3,AS4,AS5,AS10 | chr7 | 105133270 | PUS7 | chr8 | 73095161 | None | 101 | 65 |
| 4 | AS3,AS5,AS7,AS10 | chr7 | 134901829 | None | chrX | 112537121 | LOC101928437 | 108 | 35 |
| 2 | AS5,AS8 | chr7 | 142475377 | TRB | chr9 | 33802794 | LOC101929665 | 104 | 81 |
| 4 | AS1,AS5,AS7,AS10 | chr7 | 148102947 | CNTNAP2 | chr17 | 21522349 | None | 67 | 173 |
| 4 | AS5,AS8,AS9,AS10 | chr7 | 151982283 | KMT2C | chr21 | 11069677 | BAGE2 | 140 | 187 |
